# HAPLN1 confers multiple myeloma cell resistance to several classes of therapeutic drugs

**DOI:** 10.1371/journal.pone.0274704

**Published:** 2022-12-08

**Authors:** Mailee Huynh, Hae Yeun Chang, Dominique N. Lisiero, Irene M. Ong, Trinayan Kashyap, Natalie S. Callander, Shigeki Miyamoto

**Affiliations:** 1 Department of Oncology, University of Wisconsin-Madison School of Medicine and Public Health, Madison, WI, United States of America; 2 McArdle Laboratory for Cancer Research, Madison, WI, United States of America; 3 Department of Biostatistics and Medical Informatics, University of Wisconsin-Madison School of Medicine and Public Health, Madison, WI, United States of America; 4 University of Wisconsin Carbone Cancer Center (UWCCC), Madison, WI, United States of America; 5 Karyopharm Therapeutics, Inc., Newton, MA, United States of America; 6 Department of Medicine, University of Wisconsin-Madison School of Medicine and Public Health, Madison, WI, United States of America; University of Catanzaro, ITALY

## Abstract

Multiple myeloma (MM), a malignant plasma cell infiltration of the bone marrow, is generally considered incurable: resistance to multiple therapeutic drugs inevitably arises from tumor cell-intrinsic and tumor microenvironment (TME)-mediated mechanisms. Here we report that the proteoglycan tandem repeat 1 (PTR1) domain of the TME matrix protein, hyaluronan and proteoglycan link protein 1 (HAPLN1), induces a host of cell survival genes in MM cells and variable resistance to different classes of clinical drugs, including certain proteasome inhibitors, steroids, immunomodulatory drugs, and DNA damaging agents, in several MM cell lines tested. Collectively, our study identifies HAPLN1 as an extracellular matrix factor that can simultaneously confer MM cell resistance to multiple therapeutic drugs.

## Introduction

Multiple myeloma (MM) is a hematopoietic malignancy characterized by the unrestrained proliferation and accumulation of antibody secreting plasma cells in the bone marrow [1]. In the United States, MM represents ~11% of all hematological cancers and is increasing in incidence in the US (e.g., 14,400 in 1996 to 34,920 estimated in 2021) [2]. Since the first documented case of MM in 1844, the treatments available for MM have improved considerably [3], from early success with L-phenylalanine mustard (melphalan), an alkylating agent [4, 5] with addition of a corticosteroid, such as prednisone and dexamethasone [6] to highly active, more targeted agents such as the immunomodulatory drugs (IMiDs; thalidomide, lenalidomide and pomalidomide) [7–10], proteasome inhibitors (PIs; bortezomib, ixazomib, and carfilzomib) [11–13], and monoclonal antibodies (daratumumab, elotuzumab and isatuximab). Other agents, including alkylating agents (cyclophosphamide) and DNA-damaging agents (doxorubicin and bendamustine), are also employed at times in MM therapy. More recently, a selective inhibitor of nuclear export (XPO1), selinexor in combination with dexamethasone, demonstrated efficacy in MM patients previously resistant to five classes of therapies (penta-refractory) [14–17]. Drugs under development include novel agents such as cereblon E3 ligase modulator (CELMoD) such as iberdomide, the BCL2 inhibitor (venetoclax) and bispecific T cell engagers [18–20]. Finally, the recently FDA approved chimeric antigen receptor (CAR)-T cell therapy against B cell maturation antigen (BCMA) has shown promising results in relapsed/refractory MM patients. Yet patients continue to relapse [21]. Despite the vast array of currently available therapies, MM is still generally considered incurable with a median survival of 5–7 years after diagnosis. Therefore, there is a critical need to understand factors that contribute to therapy resistance and key signaling and regulatory pathways involved in therapy resistance to improve clinical outcomes [1, 3, 17, 22].

Data from sequencing efforts confirmed that MM is a highly genetically heterogeneous disease. Often observed are aberrant genetic changes in critical pathways such as: nuclear factor-kappaB (NF-κB), β-catenin, insulin-like growth factor receptor (IGFR), mitogen-activated protein kinases (MAPK), AKT, KRAS, JAK/STAT and many more [23–25]. Alterations of these various pathways to induce their constitutive activation or hyperactivation is often implicated in mediating MM cell survival and drug resistance. The complex bone marrow (BM) tumor microenvironment (TME) is also an essential component of MM pathogenesis and has been the focus of intense research efforts. It is recognized that direct and indirect interactions with different cell types, such as bone marrow stromal cells (BMSCs) and the extracellular matrix (ECM) augment MM cell growth, survival, migration, and drug resistance [26–28]. Drug resistance (DR) through the TME can be divided by two major subgroups: soluble factor mediated (SFM-DR) and cell adhesion mediated (CAM-DR). SFM-DR involves soluble cell-derived cytokines, growth factors and chemokines that act on MM cells to promote growth and survival [28]. CAM-DR is dependent on the adhesive contacts of MM cells directly to BMSCs or ECM proteins. The adhesion of MM cells through molecules, such as VLA-4 or CD44, and direct contact of myeloma cells to BMSCs allow MM cells to survive the cytotoxic effects of anti-cancer therapy [28]. Like cell-intrinsic genetic alterations, SFM-DR and CAM-DR commonly deregulate key survival signaling pathways in MM cells. Numerous ECM proteins and glycosaminoglycans (GAGs) undergo enzymatic cleavage resulting in the release of peptides (matrikines) that exert biological activities, which are usually different from those of the full-length molecules. The liberated matrikines may interact with specific receptors on the cell surface to activate several signaling pathways leading to increased migration, proliferation, or cell adhesion [29, 30]. However, whether matrikines cause drug resistance in MM remains obscure.

We previously reported that HAPLN1 is secreted by patient-derived bone marrow stromal cells, and HAPLN1 fragments containing proteoglycan tandem repeat 1 and 2 (PTR1/2) are present in MM patient bone marrow plasma or soluble fraction [31]. HAPLN1-PTR1 is sufficient to activate bortezomib-resistant NF-κB activity and confer bortezomib-resistance survival of MM cells [31]. Since NF-κB pathway plays a pivotal role in MM biology, there has been a persistent attempt to target NF-κB signaling and its effectors in anti-myeloma therapy, such as monoclonal antibodies against BAFF and BCMA, and small molecule inhibitors against NIK and IKK, among others [32]. In addition, blockade of PD-L1, a downstream effector of NF-κB signaling, is also heavily investigated [33, 34]. Whether HAPLN1-PTR1 can also induce resistance to other MM therapeutic drugs remain untested.

In this study, we report that HAPLN1-PTR1 may induce a constellation of survival genes, and confer MM cell resistance to multiple classes of therapeutic agents used in MM treatment. Our study reveals a novel mechanism of matrikine-mediated drug resistance in MM, which could represent a novel biomarker and/or therapeutic target for MM disease.

## Material and methods

### Cell line culture

RPMI8226, MM.1S, NCI-H929, and U266 human MM cell lines were obtained from American Type Culture Collection (ATCC). L363 cell line was obtained from Dr. Lixin Rui. All MM cell lines were cultured at 37°C/5% CO_2_ in RPMI1640 containing 10% FBS, 2% glutamax (Gibco) + 1% penicillin/streptomycin. These cells were checked for mycoplasma contamination by Universal Mycoplasma Detection Kit (30-1012K, ATCC) and confirmed to be negative.

### Antibodies and reagents

Antibodies against IκBα (C-21), IκBβ (C-20) were obtained from Santa Cruz Biotechnology and antibody against tubulin (CP06) purchased from Calbiochem, recombinant human TNFα (654205, EMD Millipore), cycloheximide (C7698, Sigma-Aldrich), and leptomycin B (87081-35-4, Cayman Chemicals). Bortezomib (S1013), ixazomib citrate (S2181), carfilzomib (S2853), melphalan (S8266), and bendamustine (S1212) were purchased from Selleckchem. Dexamethasone (D4902), doxorubicin (D1515), and lenalidomide (CDS022536) were purchased from Sigma-Aldrich. Selinexor (KPT-330) was provided by Dr. Trinayan Kashyap (Karyopharm). MTT (3-(4,5-dimethylthiazol-2-yl)-2,5-diphenyltetrazolium bromide) (M6494) was purchased from Thermo-Fisher Scientific.

### Purification and expression of GST-tagged proteins

The details of GST-fused HAPLN1-PTR1 and assessment of bacterial LPS contamination have been published [35]. Briefly, pGEX6p-1 plasmid-based expression constructs were transformed into BL21 Rosetta 2 *Escherichia coli* strain and induced with 1 mM IPTG followed by lysis and purification by glutathione-agarose beads (G4510, Pierce) and elution by 50 mM reduced glutathione at pH 8.0.

### Electrophoretic mobility shift assays (EMSA)

EMSA to measure NF-κB activity in MM cell lines were performed as previously described [36]. Briefly, cell extracts were made using TOTEX buffer, as previously described [37] and 10 μg of extracts were separated on 4% native polyacrylamide gel, dried, and exposed to phosphor screen (Amersham Biosciences) followed by quantitation of NF-κB DNA-binding through ImageQuant software (GE Healthcare). Each NF-κB lane was normalized to Oct-1 values from the same sample and then to the vehicle treated control values for each experiment to derive fold induction.

### SDS-PAGE and immunoblot (IB) analysis

Myeloma cell lines were pelleted and lysed in TOTEX buffer, as previously described [37]. Equal amounts (100 μg) of soluble protein were run on denaturing 10 or 12.5% SDS-PAGE gel and transferred onto a polyvinylidene fluoride or nitrocellulose membrane (GE Healthcare). The membrane was then incubated with the appropriate antibodies as described previously [37]. Immunoblots were analyzed by enhanced chemiluminescence as described by the manufacturer (GE Healthcare).

### Immunofluorescence and ImageStream cytometry

For immunofluorescence, IκBα antibody (E130, Abcam), 5 mM DRAQ5 (564902, BD Biosciences), anti-rabbit IgG (H+L), F(ab’)2 fragment (Alexa Fluor® 488 Conjugate), and viability stain (Fixable Viability Dye eFluor 780) were purchased from Thermo-Fisher Scientific. RPMI8226 cells (2 x 10^6^) were treated with or without 10 μM selinexor for 45 min at 37°C. Cells were stained with fixable viability dye eF780 for 15 min at room temperature and subsequently fixed with 4% paraformaldehyde for 10 min. eBioscience Foxp3 transcription factor staining buffer set (Thermo-Fisher Scientific) was used to permeabilize and stain cells with IκBα antibody (1:50), then F(ab’)2 Fragment (1:1000) in permeabilization buffer with 1.5% goat serum for 45 min. Nuclear DNA was labeled with DRAQ5 (1:1000) immediately preceding acquisition on the Imagestream X Mark II Imaging Flow Cytometer. Images were acquired based on circularity (Area vs. Aspect Ratio in the brightfield channel) and exclusion of the eF780 fixable viability dye. Following acquisition, IDEAS software was used to measure the degree of IκBα nuclear translocation. Similarity scores were calculated for at least 1,000 cells. The similarity score is a log-transformed Pearson’s correlation coefficient between the pixel values of two image pairs to measure the pixel intensity correlation between the IκBα and DRAQ5 images and gives the degree to which the IκBα signal is localized to the nucleus.

### RNA-sequencing

Total RNA was isolated from RPMI8226 cells treated with GST-PTR1 or GST control for 6 hours in 3 biological replicates following standard protocol with TRIzol reagent (15596–018, Thermo-Fisher). Full RNA-Seq workflow service (including library preparation, sequencing, and data QC) was provided by ProteinCT Biotechnologies (Madison, WI).

### GSEA analysis using KEGG pathways of RNA-Seq data

Gene set enrichment analysis (GSEA) was performed by calculating a ranked vector as sign(FoldChange)*1/p-value and submitted to the GSEA Ranked. The statistical significance was determined by 1,000 gene set permutations [38]. The latest KEGG pathway [39] database was used for the GSEA analysis. Analyses and plots were performed and generated using R statistical package.

### Quantitative RT-PCR (qRT-PCR) analysis

Total RNAs from treated cells were purified by a Nucleospin RNA II column (740955, Clontech) according to the manufacturer’s instruction. cDNAs were synthesized from the total RNAs using iScript cDNA synthesis kit (1708891, Bio-Rad). qRT-PCR was performed and analyzed using a Bio-Rad CFX Connect real-time system. Relative expression was determined by ΔΔCt calculation. The mRNA levels of the samples were normalized to GAPDH mRNA levels and shown as fold induction relative to GST-treated control samples. The primers for qRT-PCR analysis are: IL-8 (forward, 5’-tgcagctctgtgtgaagg-3’; reverse, 5’-ctcagccctcttcaaaaac-3’), IL-10 (forward, 5’-aggatcagctggacaacttg-3’; reverse, 5’-gatgtctgggtcttggttctc-3’), BCL2A1 (forward, 5’-tacaggctggctcaggactat-3’; reverse, 5’-cgcaacattttgtagcactctg-3’), Bcl-2 (forward, 5’-ggtggggtcatgtgtgtgg-3’; reverse, 5’-cggttcaggtactcagtcatcc-3’), and Bcl-XL (forward, 5’-gagctggtggttgactttctc-3’; reverse, 5’-tccatctccgattcagtccct-3’).

### Cell viability assay

For the MTT assay, 10^4^−10^5^ cells per well were plated in triplicate in a 96-well clear bottom microassay plate and assayed per manufacturer’s instructions. Cells containing formazan (MTT) were dissolved in 50–75 μL DMSO and formazan concentration was measured by absorbance at 540 nm. For the 10-day cell viability assessment, 5 × 10^4^ cells per well were plated in a 24-well plate and cultured for 10 days. Trypan blue assay was carried out every 2 days to assess the effect of lenalidomide on cells. The number of viable cells were counted using hemocytometer every 2 days. The assays were run in technical triplicate with GST-PTR1 treatment and the data were normalized to mean of GST control. Then, the means of three independent biological replicates were averaged to determine the mean and SD reported. The following concentrations of drugs (Table 1) were used:

**Table 1:** Drug concentrations for cell viability assay.

	RPMI8226	MM.1S	H929	L363	U266
**Bortezomib**	10 nM	10 nM	10 nM	10 nM	10 nM
**Ixazomib**	100 nM	100 nM	100 nM	100 nM	100 nM
**Carfilzomib**	100 nM	100 nM	100 nM	100 nM	100 nM
**Dexamethasone**	100 μM	100 μM	100 μM	100 μM	100 μM
**Melphalan**	10 μM	10 μM	10 μM	10 μM	10 μM
**Doxorubicin**	2 μM	0.5 μM	2 μM	2 μM	3 μM
**Bendamustine**	250 μM	50 μM	250 μM	250 μM	250 μM
**Lenalidomide**	1 μM	1 μM	1 μM	1 μM	1 μM
**Selinexor**	100 nM	100 nM	100 nM	100 nM	100 nM

### Flow cytometric analysis of rhodamine 123 efflux

Rhodamine 123 (Rh123) was obtained from Abcam (ab275545); propidium iodide (Pl) from Thermo-Fisher Scientific (ICN19545825). RPMI8226 cells pretreated with 100 nM GST-PTR1 or GST for 24 hr were stained with rhodamine 123 (10 nM) at 37°C for 60 min. Samples were analyzed by flow cytometry. The ratio of rhodamine 123 mean fluorescent intensity (MFI) was assessed.

### Statistical analysis

Unpaired two-sided Student’s t-test was used to compare two independent groups. Two-way ANOVA analysis with multiple comparisons test was used to compare PI curves and IκB degradation curves. A p-value of <0.05 was considered statistically significant. Analysis was performed with GraphPad Prism Software (GraphPad Software Inc.).

## Results

### HAPLN1-PTR1 induces a host of cell survival genes in RPMI8226 MM cells

**Fig 1:**
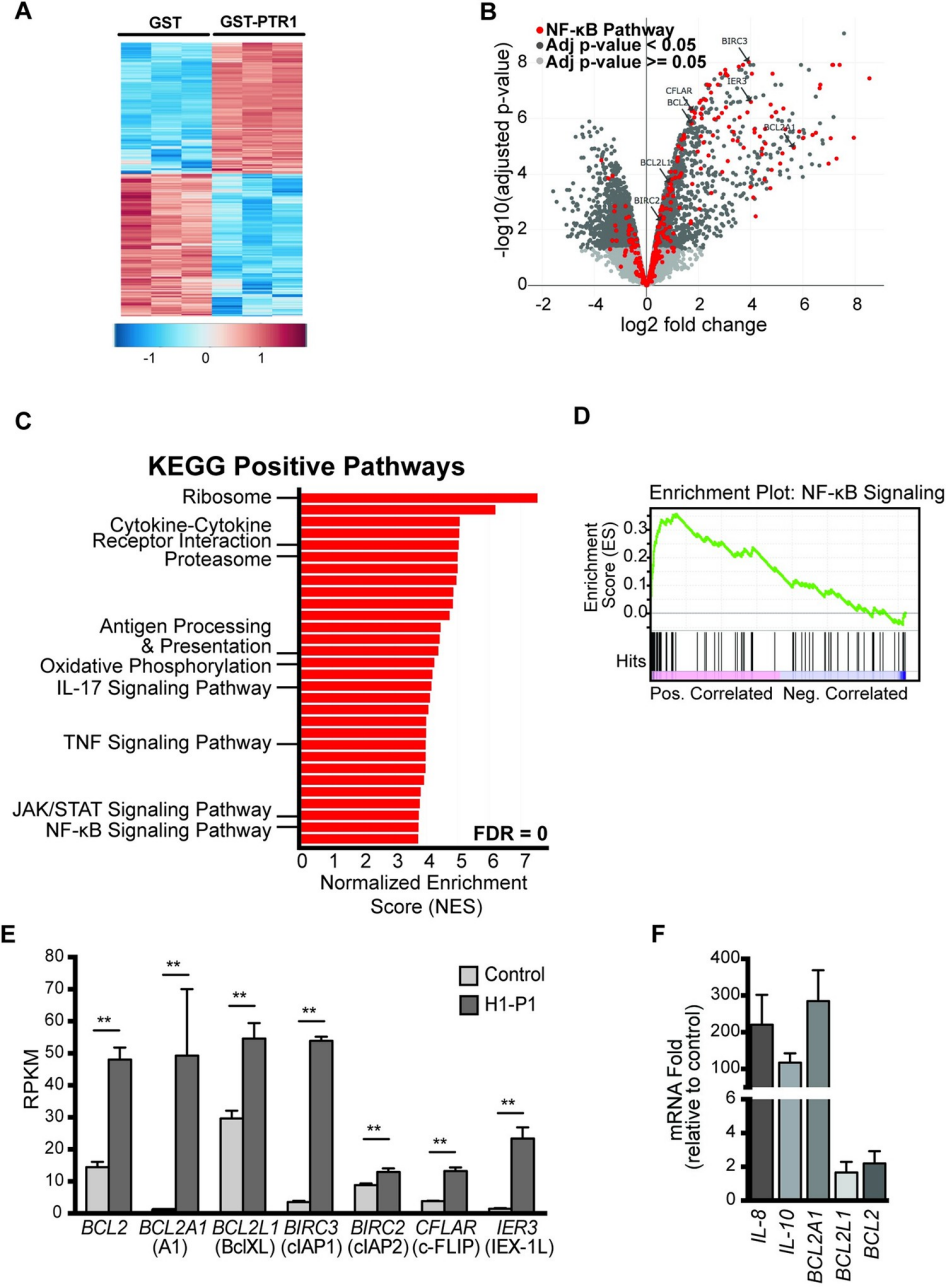
HAPLN1-PTR1 induces transcriptional changes, including an NF-κB transcriptional program. (A) Heatmap summarized all the differentially expressed genes of two-fold. Each row is a gene, each column is a sample. (B) Volcano plot of RNA-Seq data illustrating significantly (dark gray) and non-significantly (light gray) changed genes in RPMI8226 cells treated with 100 nM GST-PTR1 for 6 hr, relative to control (100 nM GST). Genes depicted in red indicate known NF-κB regulated genes. The RNA-seq analysis was done in three biological replicates. (C) Gene set enrichment analysis (GSEA) for indicated KEGG pathways and the genes differentially regulated by HAPLN1-PTR1 versus control (GST). Shown are bars indicating normalized enrichment scores (NES) for top 30 positive pathways, all with an FDR = 0. The identities of some of the pathways are indicated. For more details, see S1 Table. (D) GSEA normalized enrichment score (NES) plots of the signature of the NF-κB pathway. (E) Reads per kilobase million (RPKM) values of select NF-κB target survival genes from the RNA-Seq results in A. (F) qRT-PCR analysis of select genes detected by RNA-Seq analysis in A. RNA levels of indicated genes in GST-PTR1-treated condition were normalized to GAPDH and fold change relative to control (100 nM GST) for each gene is plotted. Results represent the mean ± SD of three biological replicates. ** p<0.01.

We previously demonstrated that the PTR1 domain of HAPLN1 (HAPLN1-PTR1), produced as recombinant glutathione-S-transferase (GST) fusion (GST-PTR1), induces pro-survival NF-κB signaling in a manner resistant to the PI bortezomib and confers resistance to this clinical drug in several human MM cell lines tested relative to GST negative control [31]. To gain insights into global transcriptional changes induced by HAPLN1-PTR1 in MM cells, we performed RNA-Seq analysis of RPMI8226 cells exposed to 100 nM GST-PTR1 for 6 hr using GST exposed cells as a negative control (GSE202672). We measured bacterial LPS contamination using highly sensitive Limulus Amebocyte Lysate assay and confirmed that it was a few orders of magnitude below that which is detectable by NF-κB EMSA assay in these cells as before [31]. We performed RNA-Seq analysis in three biological replicates, which clustered well for upregulated and downregulated genes (Fig 1A), and found that HAPLN1-PTR1 reproducibly induced ≥2-fold changes in mRNA levels of >1400 genes (Fig 1B). As expected, many NF-κB regulated genes defined by Staudt et al. [40] were detected in the GST-PTR1-upregulated gene category when compared to GST control (highlighted red in Fig 1B). Gene Set Enrichment Analysis (GSEA) [38] of GST-PTR1 RNA-Seq data identified multiple positive regulated disease pathways or phenotypes significantly correlated with GST-PTR1 treatment (Fig 1C and S1 Table) [39]. Consistent with the gene signature, GST-PTR1 treatment displayed a significant correlation to the induction of NF-κB signaling by the GSEA analysis (Fig 1D). GSEA also identified TNF signaling, JAK/STAT signaling, and immunity related changes (Fig 1C and S1 Table). Notably, NF-κB regulated genes included a host of anti-apoptotic genes, such as *BCL2*, *BCL2L1*, *BCL2A*, *BIRC2*, *BIRC3*, *CFLAR (c-FLIP) and IER3 (IEX-1L)* (Fig 1E), as well as certain immune cytokines, such as IL-8 and IL-10. We confirmed the induction of some of these genes by qRT-PCR analysis (e.g., *BCL2A1*, 284-fold; *IL-8*, 224-fold; and *IL-10*, 153-fold) (Fig 1F).

### HAPLN1-PTR1 induces RPMI8226 MM cell resistance to multiple therapeutic drugs

**Fig 2:**
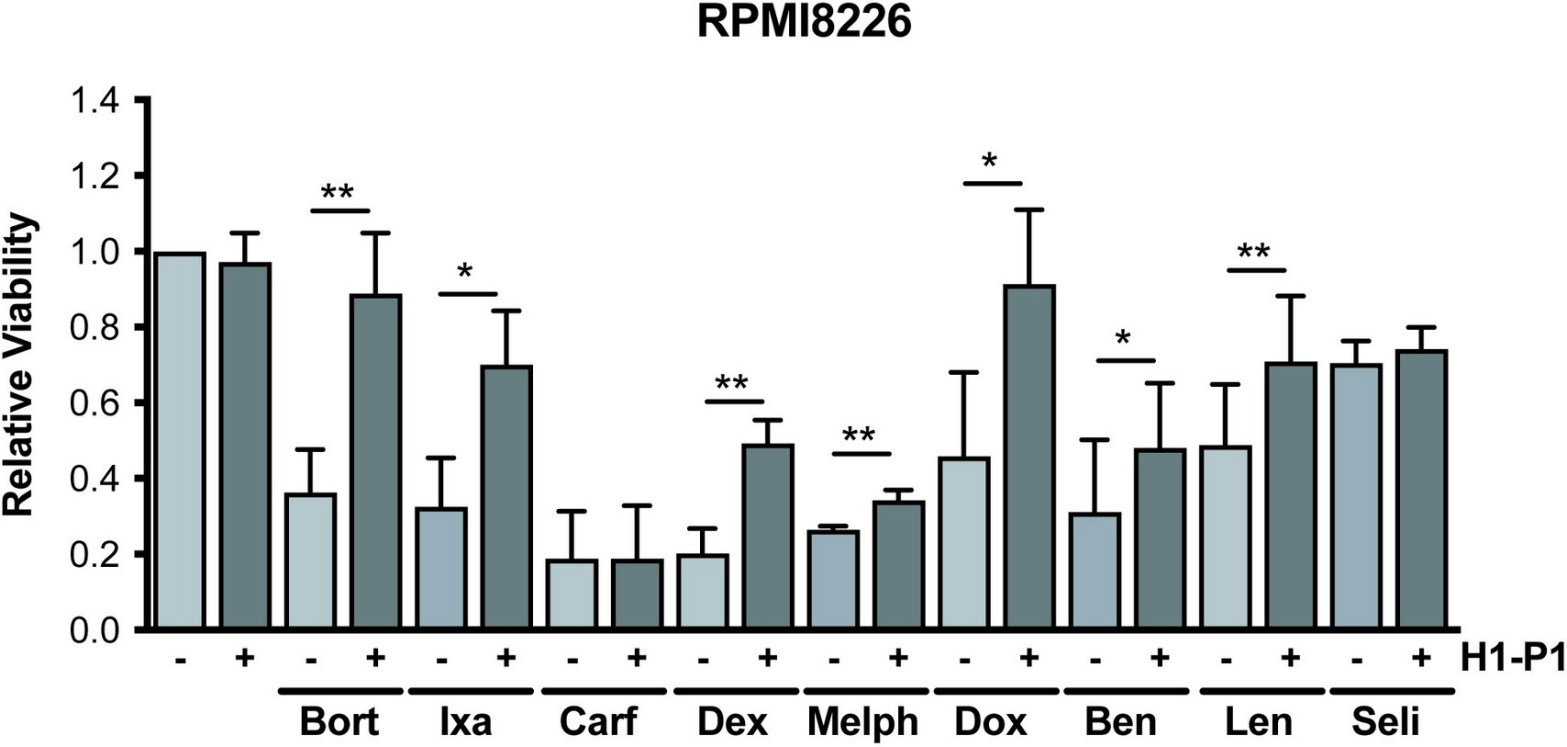
HAPLN1-PTR1 causes RPMI8226 MM cell resistance to different classes of therapeutic agents. RPMI8226 cells were cultured with 100 nM GST-PTR1 (H1-P1) or GST (-) in the presence or absence of indicated drugs at 50–80% cytotoxicity and the cell viability was measured as described in Materials and Methods. Results represent the mean ± SD of three biological replicates, each performed in triplicate. * p<0.05, ** p<0.01.

Induction of a host of anti-apoptotic genes by GST-PTR1 suggested the possibility that HAPLN1-PTR1 might induce resistance of MM cells to not only bortezomib but also other clinically employed FDA-approved drugs. To evaluate this hypothesis, we next investigated whether GST-PTR1 could increase RPMI8226 MM cell survival in the presence of different classes of FDA-approved MM therapeutics. These include other PIs (carfilzomib, ixazomib), a glucocorticoid (dexamethasone), DNA damaging agents (melphalan, doxorubicin, bendamustine), an IMiD (lenalidomide), and a nuclear export inhibitor (selinexor). We first exposed RPMI8226 cells to varying concentrations of these drugs and identified drug doses that would yield ~50–80% toxicity using MTT assays at 24-hours (PIs, doxorubicin), 2 days (bendamustine), or 3 days (dexamethasone, melphalan, selinexor), or longer 10-day growth assay (lenalidomide). IMiD-induced toxicity is known to manifest much more slowly than that induced by other cytotoxic MM drugs [41]. The cells were then treated with 100 nM GST-PTR1 or GST in the presence of these MM drugs. These toxicity assays demonstrated that GST-PTR1 caused significant resistance of RPMI8226 MM cells to all the drugs tested, except for carfilzomib and selinexor (Fig 2). GST-PTR1 alone without the drugs did not affect the growth of the MM cells. These results demonstrate that HAPLN1-PTR1 is capable of inducing RPMI8226 cell resistance to multiple MM drugs.

### HAPLN1-PTR1 induces clinical PI resistant NF-κB activation in RPMI8226 MM cells

**Fig 3:**
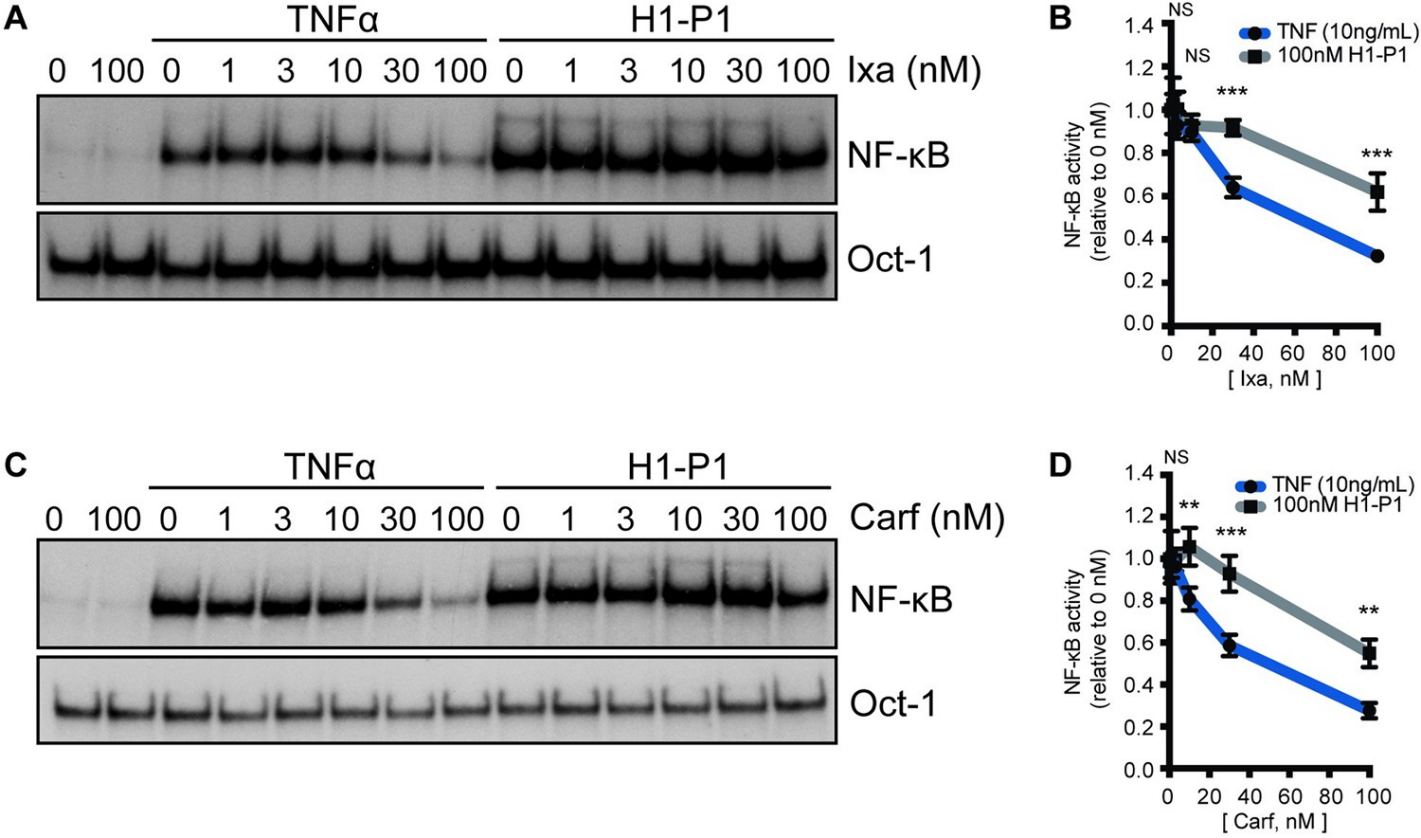
HAPLN1-PTR1-induced NF-κB activation is resistant to clinical PI’s. (A) Representative EMSA analysis of RPMI8226 cells incubated with 10 ng/mL TNFα or 100 nM GST-PTR1 (H1-P1) in the absence or presence of increasing concentrations (nM) of ixazomib (Ixa). (B) Graph depicts the mean ± SD of the quantification of three independent replicates of EMSA analysis as in A. (C) Representative EMSA analysis as in A with increasing concentrations (nM) of carfilzomib (Carf). (D) Graph depicts the mean ± SD of the quantification of three independent replicates of EMSA analysis as in C. ** p<0.01, *** p<0.001.

We previously showed that HAPLN1-PTR1 induces bortezomib-resistant NF-κB signaling, which was correlated with bortezomib resistance in RPMI8226 cells [31]. The finding that GST-PTR1 can cause resistance to ixazomib but not carfilzomib in the above study could be related to the ability of the latter to inhibit NF-κB signaling induced by HAPLN1-PTR1 but not by the former. To test this hypothesis, we treated RPMI8226 cells at different concentrations of ixazomib and carfilzomib, in the presence of GST-PTR1 or GST control. To demonstrate the efficacy of these PIs, the cells were also treated in parallel with TNFα, a canonical NF-κB inducer that involves the 26S proteasome-mediated IκBα degradation to activate NF-κB. The cell samples were then analyzed by EMSA using Igκ-κB probe with Oct-1 binding serving as a loading control. Similar to the case with bortezomib, NF-κB activation by GST-PTR1 was not inhibited by ixazomib or carfilzomib up to 30 nM (Fig 3A–3D). Significant NF-κB inhibition was only evident at 100 nM of these PIs. In contrast, significant inhibition of NF-κB activation by TNFα was evident at 30 nM of ixasomib and 10 nM for carfilzomib. The degree of inhibition was significantly greater for TNFα than that observed for GST-PTR1 at the varying PIs concentrations analyzed (Fig 3A–3D). These results suggest that, similar to bortezomib [31], NF-κB activation by HAPLN1-PTR1 is significantly more resistant to both ixazomib and carfilzomib relative to TNFα-induced activation. HAPLN1-PTR1 can reduce the MM cell toxicity of both bortezomib and ixazomib, but not carfilzomib, indicating that the latter drug can escape the resistance impact of HAPLN1-PTR1 despite its inability to inhibit NF-κB activation.

### Selinexor inhibits HAPLN1-PTR1-induced IκBα degradation and NF-κB activation

**Fig 4:**
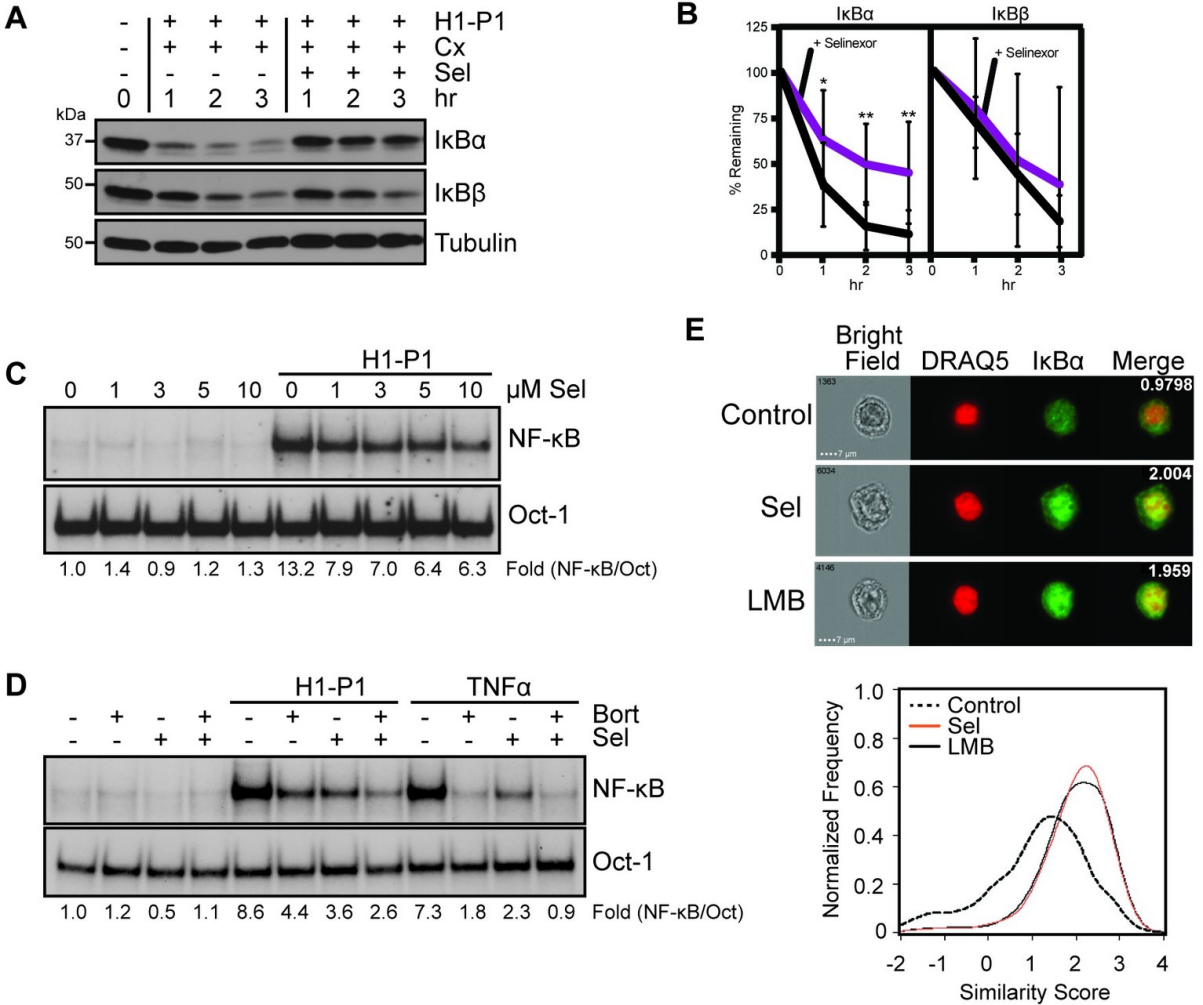
Selinexor inhibits HAPLN1-PTR1-induced IκBα degradation and NF-κB activation. (A) Representative immunoblot analysis of IκBα, IκBβ and tubulin in RPMI8226 cells pretreated for 30 min with 20 μg/mL cycloheximide (Cx) with 10 μM selinexor (Sel) and stimulated with GST-PTR1 (H1-P1) for indicated times. (B) Graph depicts the mean ± SD of the quantification of three independent replicates of western blot analysis as in A. (C) EMSA analysis of RPMI8226 cells pretreated for 30 min with increasing concentrations of selinexor (Sel) and stimulated with GST-PTR1 (H1-P1). (D) EMSA analysis of RPMI8226 cells pretreated for 30 min with 10 μM Sel or 100 nM Bort and stimulated with 100 nM GST-PTR1 (H1-P1) or 10 ng/mL TNFα. (E) Upper: Images of RPMI8226 cells control and treated with 10 μM Selinexor (Sel) or 20 μg/mL Leptomycin B (LMB) for 45 min. The similarity score for each set of representative images is included in merged image. Lower: A representative similarity histogram for control and treated cells showing the co-localization of IκBα and the nuclear dye, DRAQ5.

We previously demonstrated that NF-κB activation by HAPLN1-PTR1 involves PI-resistant degradation of IκBα and PI-sensitive IκBβ degradation [31]. Of these, inhibition of IκBα degradation by selinexor was reported to be critical for its clinical efficacy [42]. We and others also reported that IκBα, but not IκBβ, contains a nuclear export sequence (NES), critical for nuclear export of inactive NF-κB-IκBα complexes to the cytoplasm via the nuclear export receptor XPO1/CRM1 [43, 44]. We further reported that the nuclear export inhibitor Leptomycin B (LMB) or a mutation of IκBα-NES can prevent PI resistant IκBα degradation via nuclear sequestration and inhibit atypical bortezomib-resistant NF-κB activity [45]. Because both selinexor and LMB are specific chemically distinct inhibitors of XPO1, we tested whether the ability of selinexor to overcome HAPLN1-PTR1-mediated drug resistance was related to its ability to prevent HAPLN1-PTR1-induced IκBα degradation and associated NF-κB activation. RPMI8226 cells were exposed to GST-PTR1 in the presence of cycloheximide to block new IκBα synthesis to specifically determine the impact of its degradation induced by the ligand with and without selinexor. We previously demonstrated that IκBα levels are not altered by three-hour treatment with cycloheximide in RPMI8226 cells [31]. GST-PTR1 induced near complete degradation of IκBα within 1 hour, which was inhibited by selinexor (Fig 4A and 4B). This resulted in partial inhibition of NF-κB activation induced by GST-PTR1 (Fig 4C); the remaining activation is likely arising from degradation of IκBβ, which is insensitive to selinexor (Fig 4A and 4B) but sensitive to bortezomib [31]. Indeed, this is confirmed by addition of both selinexor and bortezomib that caused near complete NF-κB inhibition induced by GST-PTR1 (Fig 4D). As expected, selinexor caused nuclear accumulation of IκBα, like LMB treatment (Fig 4E). These results support the hypothesis that selinexor can block HAPLN1-PTR1-induced IκBα degradation via its nuclear sequestration causing inhibition of IκBα-associated NF-κB activation to overcome the survival effects induced by HAPLN1-PTR1 in MM cells. Moreover, these results provide additional molecular details potentially underlying the marked clinical synergy with the combination of selinexor and PIs [46].

### HAPLN1-PTR1 also induces expression of *multi-drug resistance (MDR)* genes and function in RPMI8226 cells

**Fig 5:**
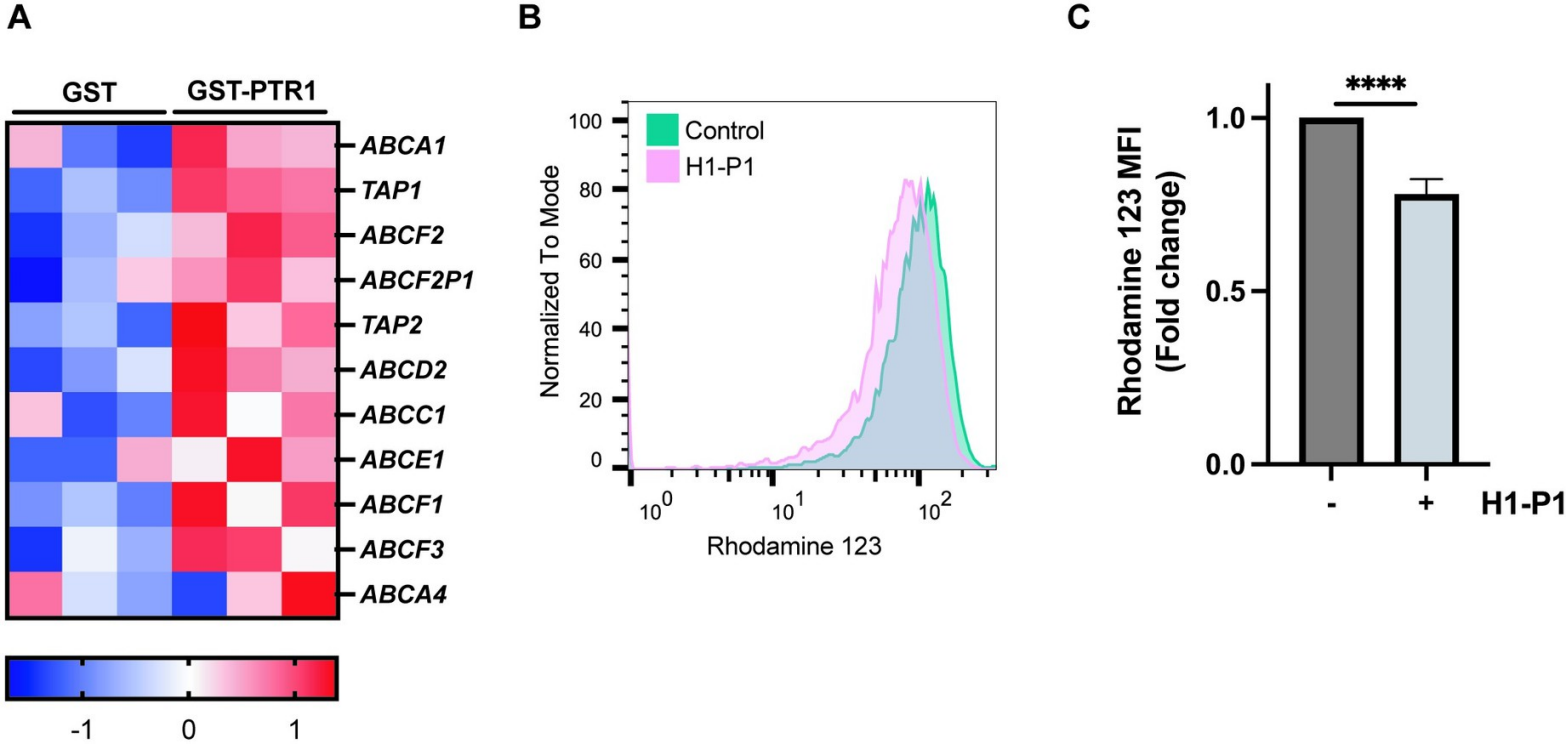
HAPLN1-PTR1 increases expression of MDR genes and function. (A) Heatmap of upregulated MDR genes after GST-PTR1 treatment in RNA-seq analysis colored by Z-score. (B) Representative histogram of rhodamine 123 efflux assay. RPMI8226 cells were treated with 100 nM GST-PTR1 (H1-P1) or control GST for 24 h and subjected to the rhodamine 123 efflux assay. (C) Graph depicts the mean fluorescent intensity (MFI) ± SEM of three independent replicates of rhodamine 123 efflux assay as in B. **** p<0.0001.

**While the above studies focused on the potential role of NF-kB-regulated antiapoptotic genes induced by HAPLN1-PTR1, our RNA-seq analysis also identified various** *MDR* **family genes were also upregulated after GST-PTR1 treatment (Fig 5A). Moreover, using the drug efflux pump substrate, rhodamine 123, GST-PTR1 also significantly upregulated MDR efflux function (Fig 5B and 5C). These results suggest a dual mode of HAPLN1-induced drug resistance mechanism by inducing the expression of both anti-apoptotic genes and drug efflux pump** *MDR* **genes.**

### HAPLN1-PTR1 induces variable levels of drug resistance in several myeloma cell lines

**Fig 6:**
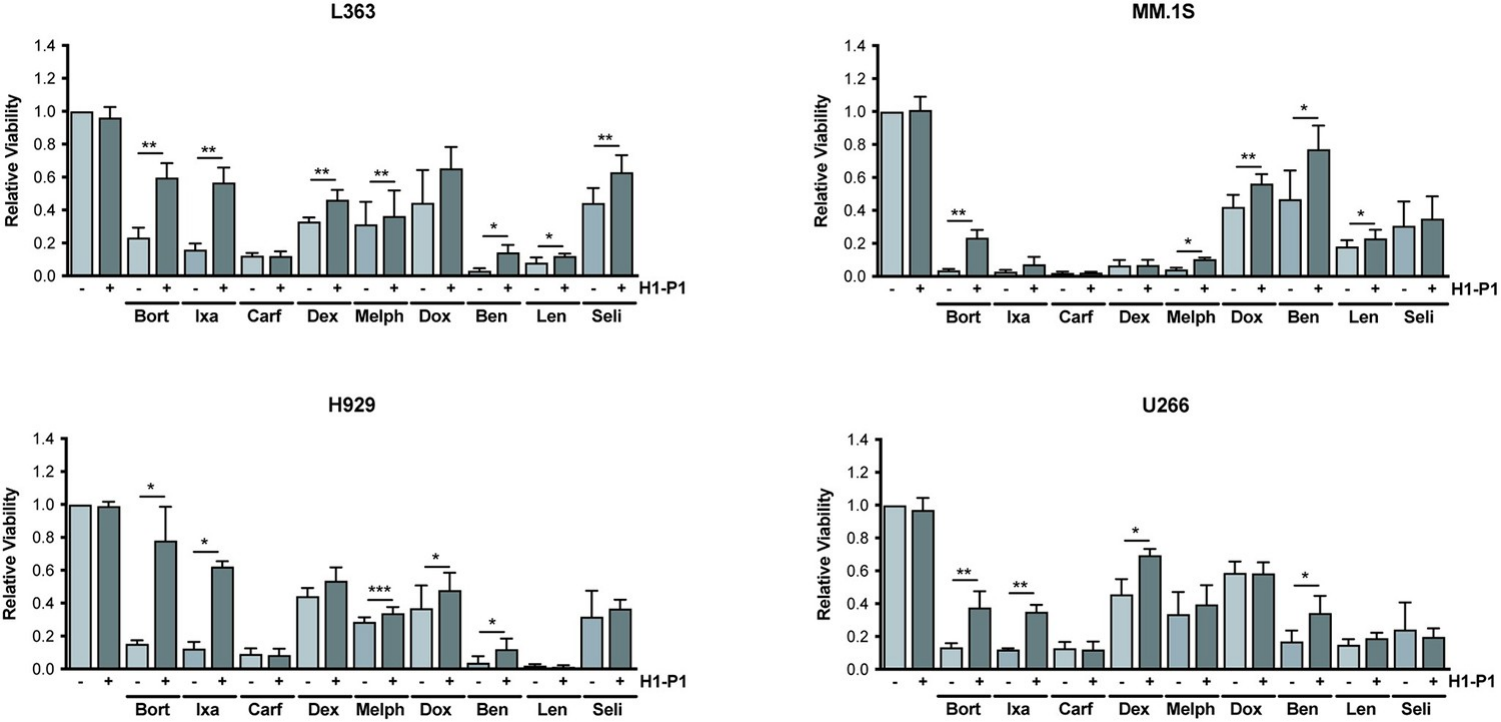
HAPLN1-PTR1 causes resistance of several MM cell lines to multiple clinical drugs. MM cell lines (L363, MM.1S, H929, U266) were cultured with 100 nM GST-PTR1 (H1-P1) or GST (-) in the presence or absence of indicated drugs and the cell viability was measured as described in Materials and Methods. Results represent the mean ± SD of three biological replicates, each performed in triplicate. * p<0.05, ** p<0.01, *** p<0.001.

To determine whether the drug resistance phenotype induced by HAPLN1-PTR1 in RPMI8226 cells is not limited to this MM cell line alone, we exposed several other MM cell lines to different clinical drugs in the presence of GST-PTR1 or GST as control. As in the RPMI8226 cell study, these studies were performed in biological triplicates, each performed in three technical replicates. The results are summarized in Fig 6, which demonstrated that GST-PTR1 was able to induce variable but significant resistance to multiple classes of MM therapeutic drugs in different MM cell lines. Again, as in the case for RPMI8226 cells, GST-PTR1 failed to induce resistance to carfilzomib in all cell lines tested and to selinexor in most lines. Overall, our results identify HAPLN1-PTR1 as a novel extracellular matrix factor capable of inducing resistance in MM cells to multiple classes of MM therapeutics with notable exceptions.

## Discussion

In the present study, we tested whether HAPLN1 acts as a drug resistance factor in MM cells against different classes of therapeutic drugs. We previously showed that HAPLN1 can initiate bortezomib-resistant NF-κB activity in MM cells and bortezomib-resistant cell survival [31]. We now performed an RNA-seq analysis of RPMI8226 MM cell line exposed to HAPLN1-PTR1 and found that this ligand induces significant changes in the transcriptomic landscape in these MM cells. This included induction of a group of anti-apoptotic genes that are known targets of NF-κB, among many other genes. Because these changes in expression of anti-apoptotic genes may increase the death threshold of MM cells, we tested whether HAPLN1-PTR1 could also increase resistance of MM cells to other clinically employed MM therapeutics. Indeed, this ligand was able to variably increase MM cell resistance to many of the currently employed drugs, including PIs, glucocorticoids, DNA damaging agents, and IMiDs, in several MM cell lines tested. HAPLN1 also induced the expression of a host of *MDR* genes and drug efflux function of MM cells. These results therefore support HAPLN1 as a novel ECM-derived multi-drug resistance inducer in MM cells.

Interestingly, HAPLN1 was found to mediate resistance to apoptosis induction by bortezomib and ixazomib, but not by carfilzomib. We found that HAPLN1-PTR1-induced NF-κB activity was highly resistant to all the PIs tested, including carfilzomib, thus further demonstrating the induction of NF-κB activation by HAPLN1 proceeds via an atypical PI-resistant mechanism. Nevertheless, HAPLN1-PTR1 was unable to cause apoptosis resistance to carfilzomib in any of the MM cell lines tested even though it did so against bortezomib and ixazomib in almost all cell lines tested. The difference in HAPLN1-mediated survival could potentially be attributed to differences in pharmacological properties of the PIs. Bortezomib and ixazomib are chemically related (peptide boronates) and are both reversible inhibitors of the proteasome [47]. In contrast, carfilzomib has a different chemical structure based on four amino acids and irreversibly binds to the proteasome via epoxyketone pharmacophore [48]. Furthermore, carfilzomib and dexamethasone regimen was shown to be clinically superior to bortezomib and dexamethasone in patients with relapsed MM in a large phase III randomized trial [49]. Indeed, NF-κB activation induced by HAPLN1-PTR1 was also resistant to oprozomib, another irreversible epoxyketone structured PI [47], but it was still unable to cause resistance to this PI similar to carfilzomib (S1 Fig). Other than sharing the similar chemical structure, carfilzomib and oprozomib differ in their selectivity against target proteasome β subunits, i.e., carfilzomib inhibits β5/2/1, oprozomib only β5 [47]. This suggests that HAPLN1 can induce drug resistance only against reversible PIs, but irreversible PIs can overcome HAPLN1-induced survival effects. It is therefore likely that prolonged proteasome inhibition, possibly only with irreversible PIs, can overcome HAPLN1 effects.

The differences in sensitivities to HAPLN1-induced drug resistance may also be due to differences in resistance mechanisms involved for different PIs. Unlike bortezomib, whose various resistance mechanisms including NF-κB signaling have been described in the literature [50–56], the mechanisms of resistance to other PIs are not well defined. It is possible that other signaling pathways induced by HAPLN1, such as the JAK/STAT pathway identified by the KEGG analysis of our RNA-seq data, might be playing a role in mediating differential effects against different PIs. In patients, carfilzomib shows clinical efficacy against MM resistant to bortezomib, thus demonstrating that this second generation PI is capable of overcoming bortezomib resistance mechanism(s) [57]. Besse et al., demonstrated that PI’s difference in the β subunit selectivity exerts the differential cytotoxic effect, namely, co-inhibition of β5/β2 subunits has the most cytotoxicity which can be achieved by high dose carfilzomib, while bortezomib can only abrogate β5/β1 [58]. Moreover, bortezomib and carfilzomib resistant MM cell lines manifest different *PSMB5* mutation status and MDR gene expression. Unlike bortezomib resistant cell lines, carfilzomib resistant MM cells have wild-type *PSMB5*, but overexpress the drug efflux pump ABCB1/MDR1 [58, 59]. Interestingly, we also observed induction of a host of MDR gene family members in response to HAPLN1 stimulation of MM cells but ABCB1/MDR1 was not among those induced. Thus, further studies are required to define the mechanism by which the irreversible PI carfilzomib overcomes HAPLN1-induced survival effects in MM cells.

Similar to carfilzomib, selinexor also overcame HAPLN1-induced survival effects in most MM cell lines analyzed. Selinexor is an oral first-in-class selective inhibitor of XPO1-mediated nuclear export (SINE) compound [42]. This inhibitor was able to abolish HAPLN1-PTR1 induced IκBα degradation by forcing nuclear retention and accumulation of IκBα. This is consistent with our previous findings that PI-resistant IκBα degradation requires the presence of IκBα in the cytoplasm and thus its nuclear accumulation induced by XPO1/CRM1 inhibitors prevents its degradation [60]. Consequently, selinexor suppressed HAPLN1-induced NF-κB activation. Prior studies have found that cytotoxic effects of selinexor against MM cells (and other cancer types) depend, at least part, on IκBα [61]. Thus, through the inhibition of IκBα nuclear export, selinexor appears to overcome the HAPLN1-induced NF-κB signaling and survival effects. Selinexor has been previously shown to inhibit NF-κB transcriptional activity in different cancer and inflammatory models [62–64]. However, selinexor also affects multiple additional key molecular targets, such as p53, p21, p27, STAT1, and STAT3 [65–68], which may also contribute to overcoming the survival effects conferred by HAPLN1.

GSEA analysis of RNA-Seq data also showed that HAPLN1 is not only linked to pathways related to drug resistance but it may have a wider role in orchestrating the intra-tumoral inflammatory milieu and balancing anti-tumor immunity. These results pointed to increased expression of pathways correlated to T cell-mediated immunity and anti-viral responses. This is intriguing in view of recent findings showing that the regulated proteolysis of the tolerogenic versican (VCAN) generates a matrikine, versikine, with opposing, immunostimulatory activities [69, 70]. VCAN proteolysis correlates with T-cell infiltration in both hematopoietic and solid tumors and versikine triggers a type-I interferon response in myeloid cells, an essential component of the “T-cell inflamed” TME [71, 72]. Notably, HAPLN1 is a critical component of VCAN-ECM and like HAPLN1, VCAN/versikine contains two related PTR-domains. Taken together, these findings point to coordinated roles of PTR-containing proteins in regulating drug resistance and tumor immunity. It seems plausible that distinct components of the MM TME contribute diverse PTR-containing matrikines. It would be of great interest to determine whether the levels of such matrikines vary through MM disease progression and whether their levels could be predictive of therapy resistance and possibly immune microenvironment. Individualized therapeutic approaches using the HAPLN1 level in patient plasma as a prognostic marker need to be further tested in well-designed clinical settings. Such an approach would require the development of an assay to quantify HAPLN1 matrikine levels in patient samples.

## Conclusions

The data presented here demonstrate that a HAPLN1 matrikine can induce resistance in MM cells to different classes of therapeutic agents. This matrikine induces many changes in cell signaling and gene expression in MM cells, including an activation of NF-κB signaling and induction of many cell survival genes, multi-drug resistance genes, and immune-related genes. Our study thus reveals a novel mechanism of drug resistance in MM cells, suggesting that the HAPLN1 matrikine may be a novel biomarker and/or therapeutic target for this currently incurable disease.

## Supporting information

S1 Table Gene set enrichment analysis (GSEA) using KEGG pathways of the ranked genes differentially regulated by GST-PTR1 versus GST control. Top 30 significant (FDR<0.05) pathways are shown. KEGG, Kyoto Encyclopedia of Genes and Genomes; ES, enrichment score; NES, normalized enrichment score; NOM, nominal; FDR, false discovery rate; FWER, family-wise error rate.
(TIF)

S1 Fig HAPLN1-PTR1-induced NF-κB activation is resistant to oprozomib. (A) Representative EMSA analysis of RPMI8226 cells incubated with 10 ng/mL TNFα for 15 min or 100 nM GST-PTR1 (H1-P1) for 2 hr in the absence or presence of increasing concentrations (nM) of oprozomib (Opro). (B) Graph depicts the mean ± SD of the quantification of three independent replicates of EMSA analysis as in A. (C) MM cell lines (RPMI8226, L363, MM.1S, H929, U266) were cultured with 100 nM GST-PTR1 (H1-P1) or GST (-) in the presence of oprozomib (200 nM) and the cell viability was measured as described in Materials and Methods. Results represent the mean ± SD of three biological replicates, each performed in triplicate. ** p<0.01.
(TIF)

S1 Raw images Original blots and gel image data. (PDF)
