# Supplementary figures and images for: HAPLN1 confers multiple myeloma cell resistance to several classes of therapeutic drugs
Source: PLoS One. 2022 Dec 8;17(12):e0274704. doi: 10.1371/journal.pone.0274704 (PMC10045543; doi:10.1371/journal.pone.0274704)

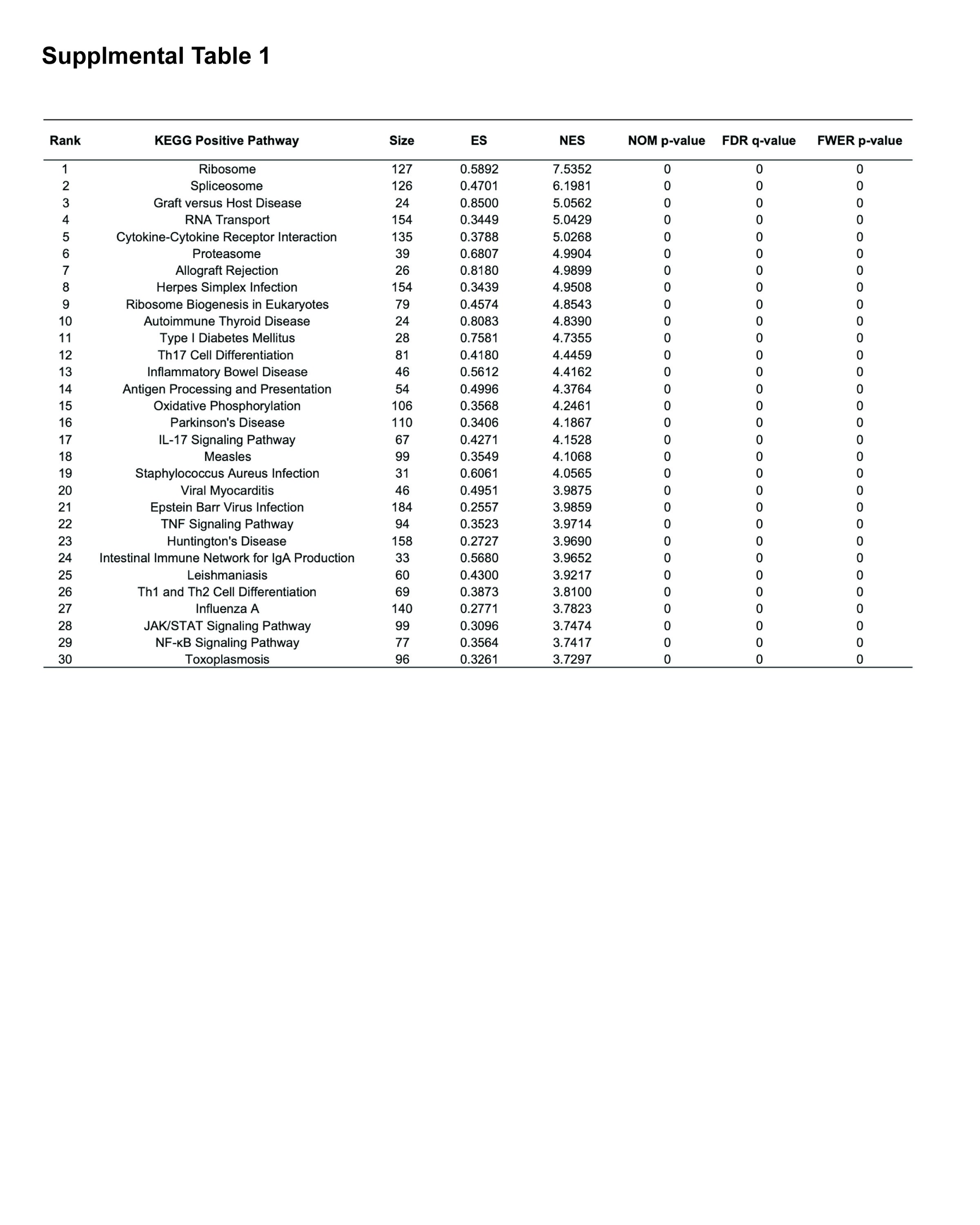

Supplement: S1 Table — Top 30 significant (FDR<0.05) pathways are shown. KEGG, Kyoto Encyclopedia of Genes and Genomes; ES, enrichment score; NES, normalized enrichment score; NOM, nominal; FDR, false discovery rate; FWER, family-wise error rate. (TIF) [file pone.0274704.s001.tif]

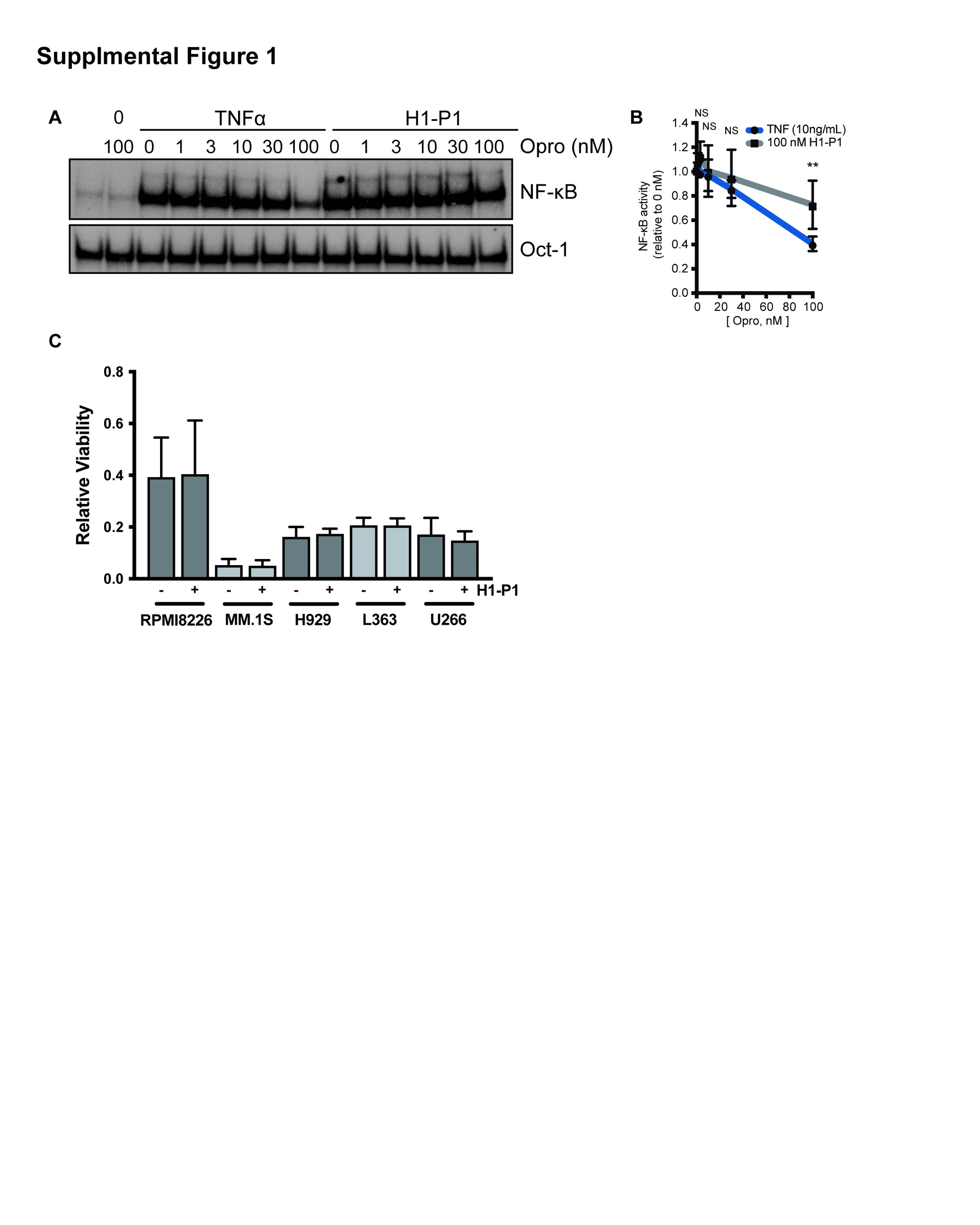

Supplement: S1 Fig — (A) Representative EMSA analysis of RPMI8226 cells incubated with 10 ng/mL TNFα for 15 min or 100 nM GST-PTR1 (H1-P1) for 2 hr in the absence or presence of increasing concentrations (nM) of oprozomib (Opro). (B) Graph depicts the mean ± SD of the quantification of three independent replicates of EMSA analysis as in A. (C) MM cell lines (RPMI8226, L363, MM.1S, H929, U266) were cultured with 100 nM GST-PTR1 (H1-P1) or GST (-) in the presence of oprozomib (200 nM) and the cell viability was measured as described in Materials and Methods. Results represent the mean ± SD of three biological replicates, each performed in triplicate. ** p<0.01. (TIF) [file pone.0274704.s002.tif]
