# Supplementary material for: HAPLN1 confers multiple myeloma cell resistance to several classes of therapeutic drugs
Source: PLoS One. 2022 Dec 8;17(12):e0274704. doi: 10.1371/journal.pone.0274704 (PMC10045543; doi:10.1371/journal.pone.0274704)

Fig 3A

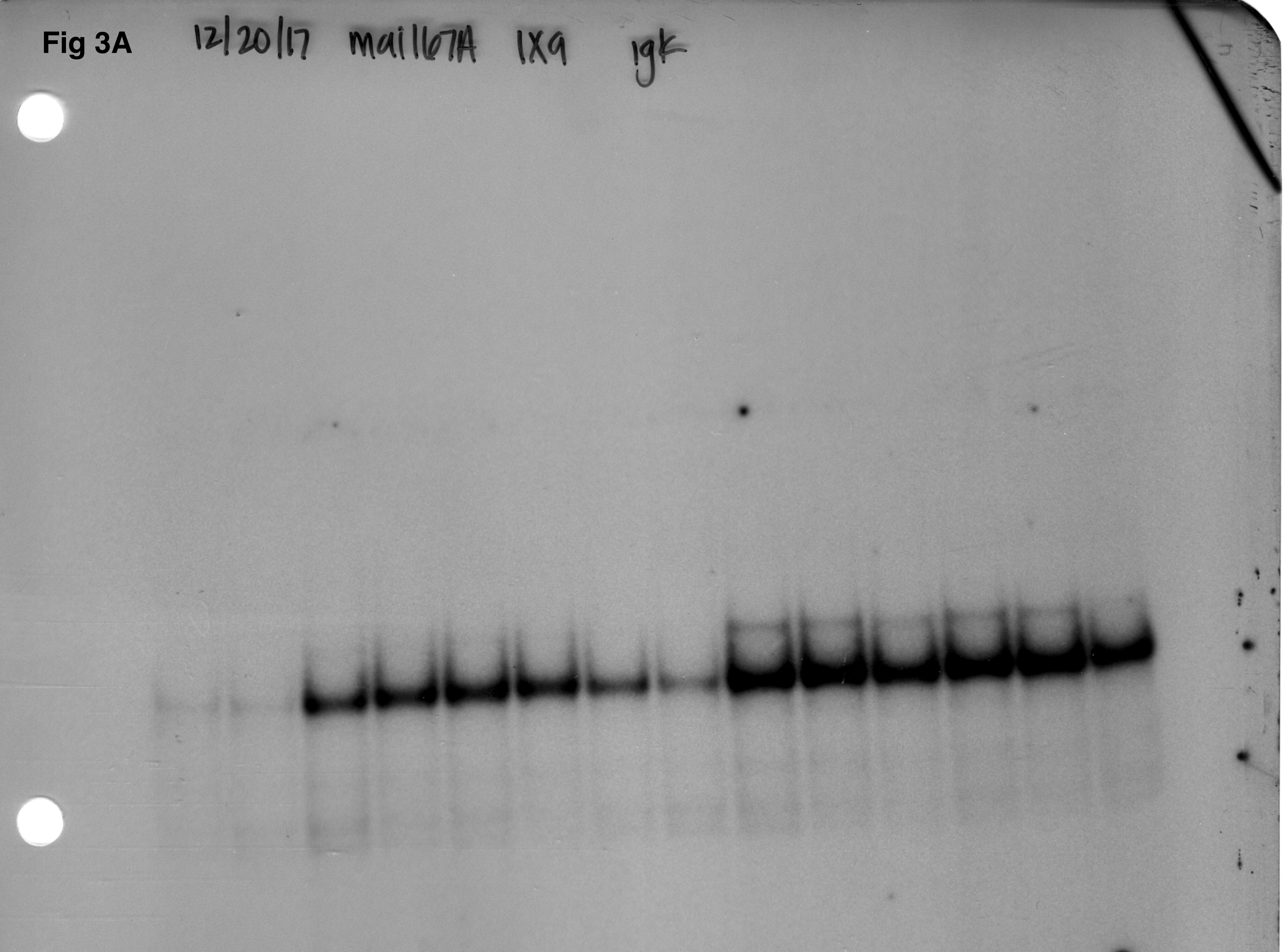

Fig 3A

12/20/17 Ma1167A 1X9

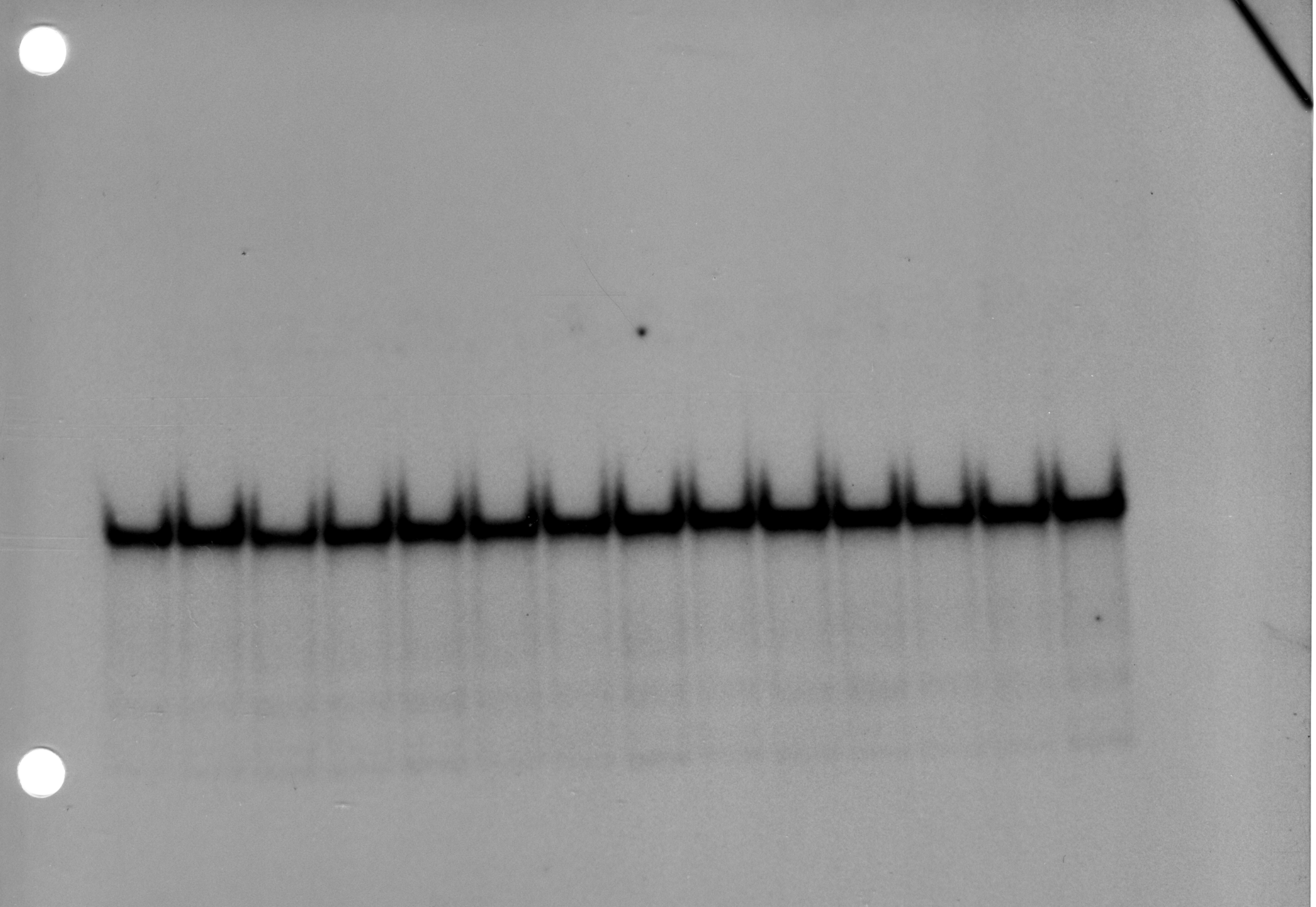

Fig 3C

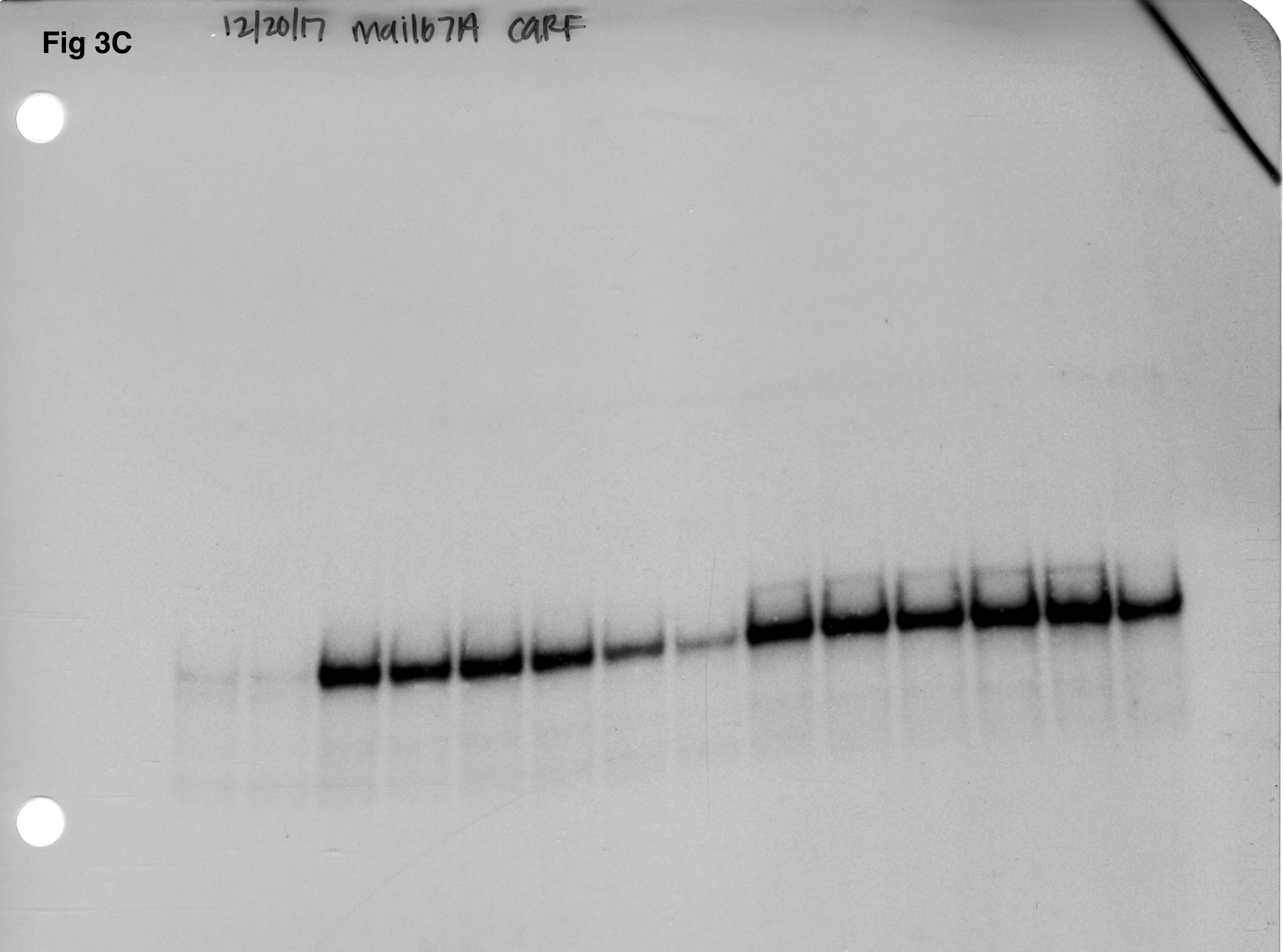

Fig 3C

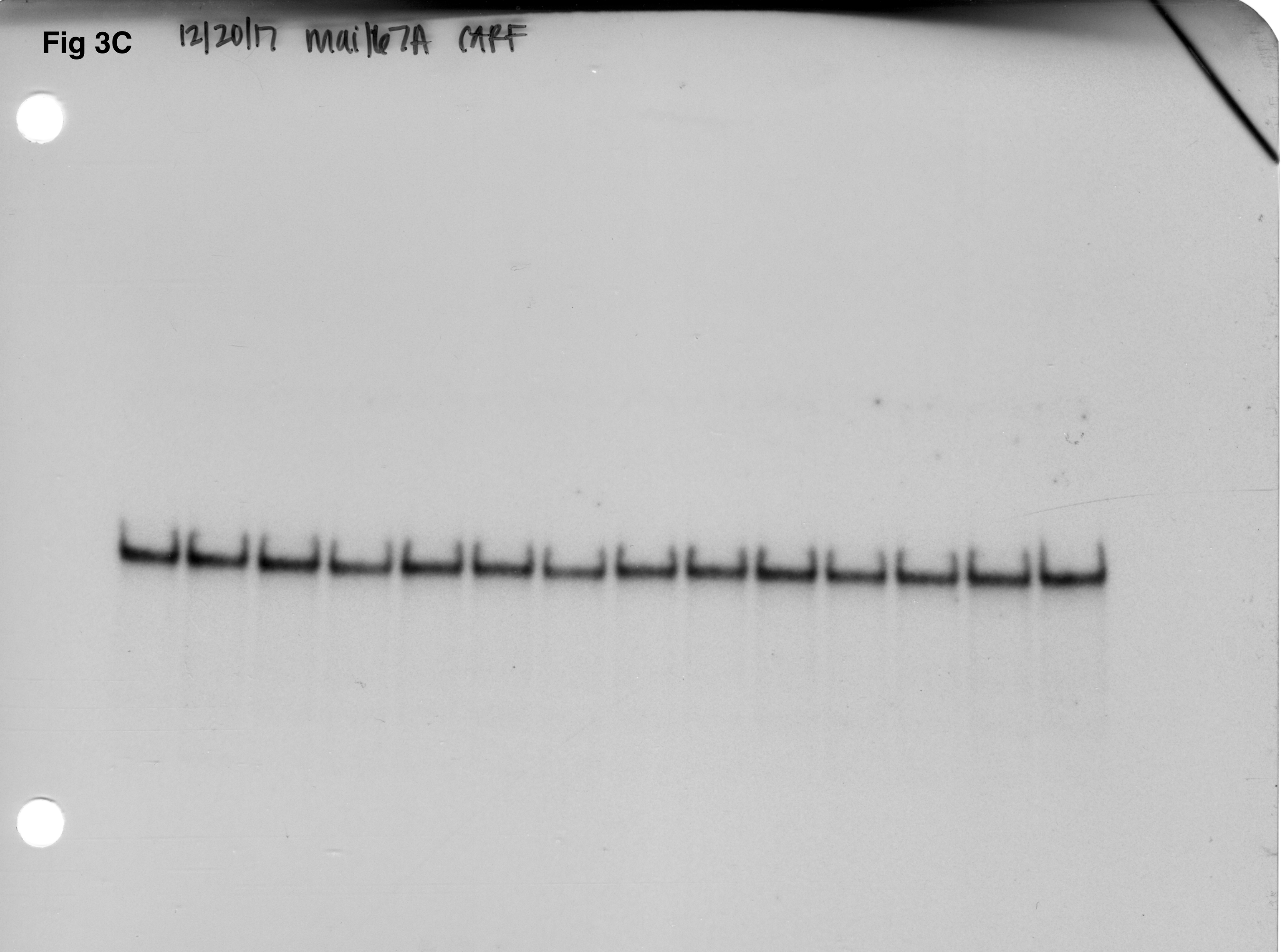

Fig 4A

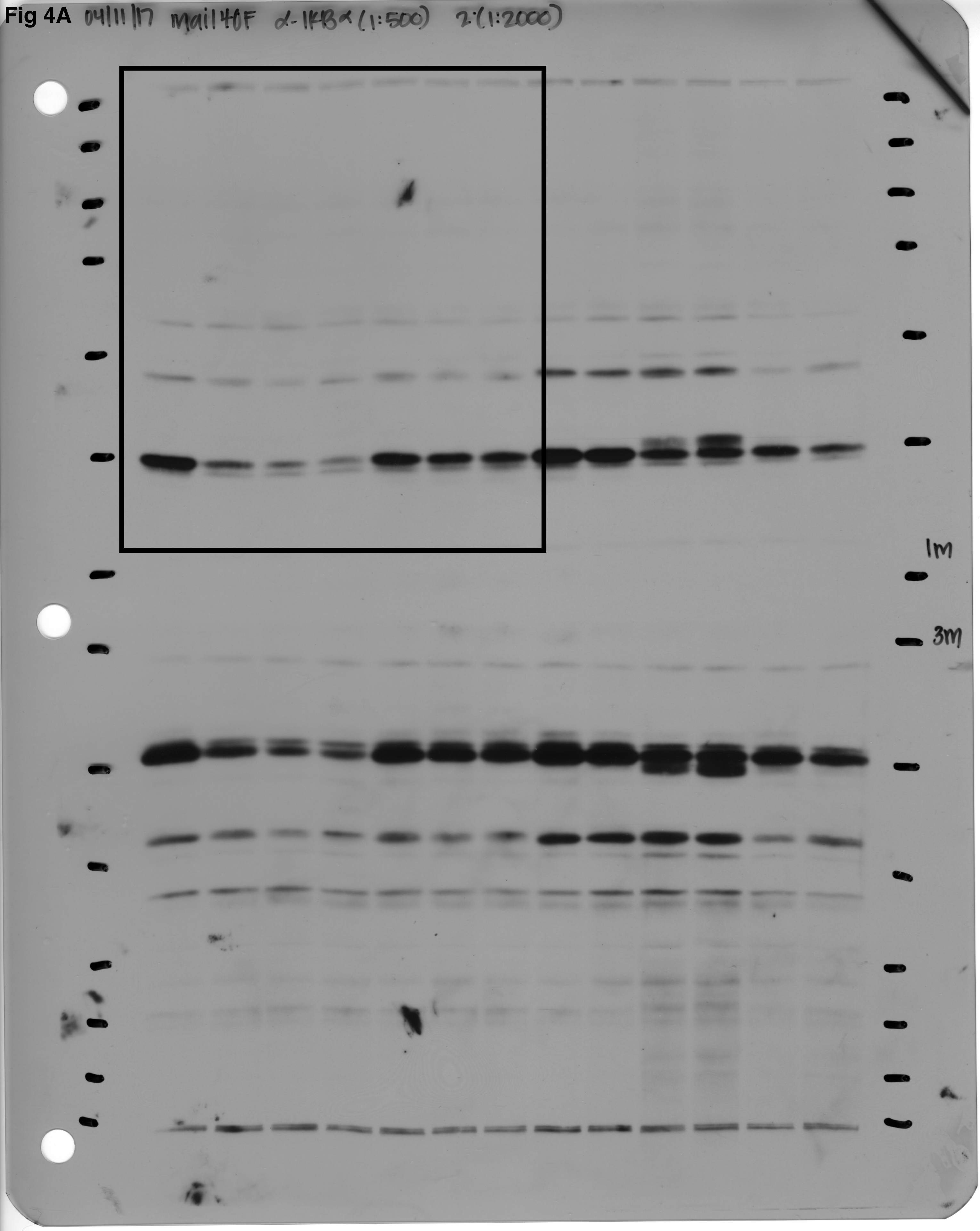

Fig 4A

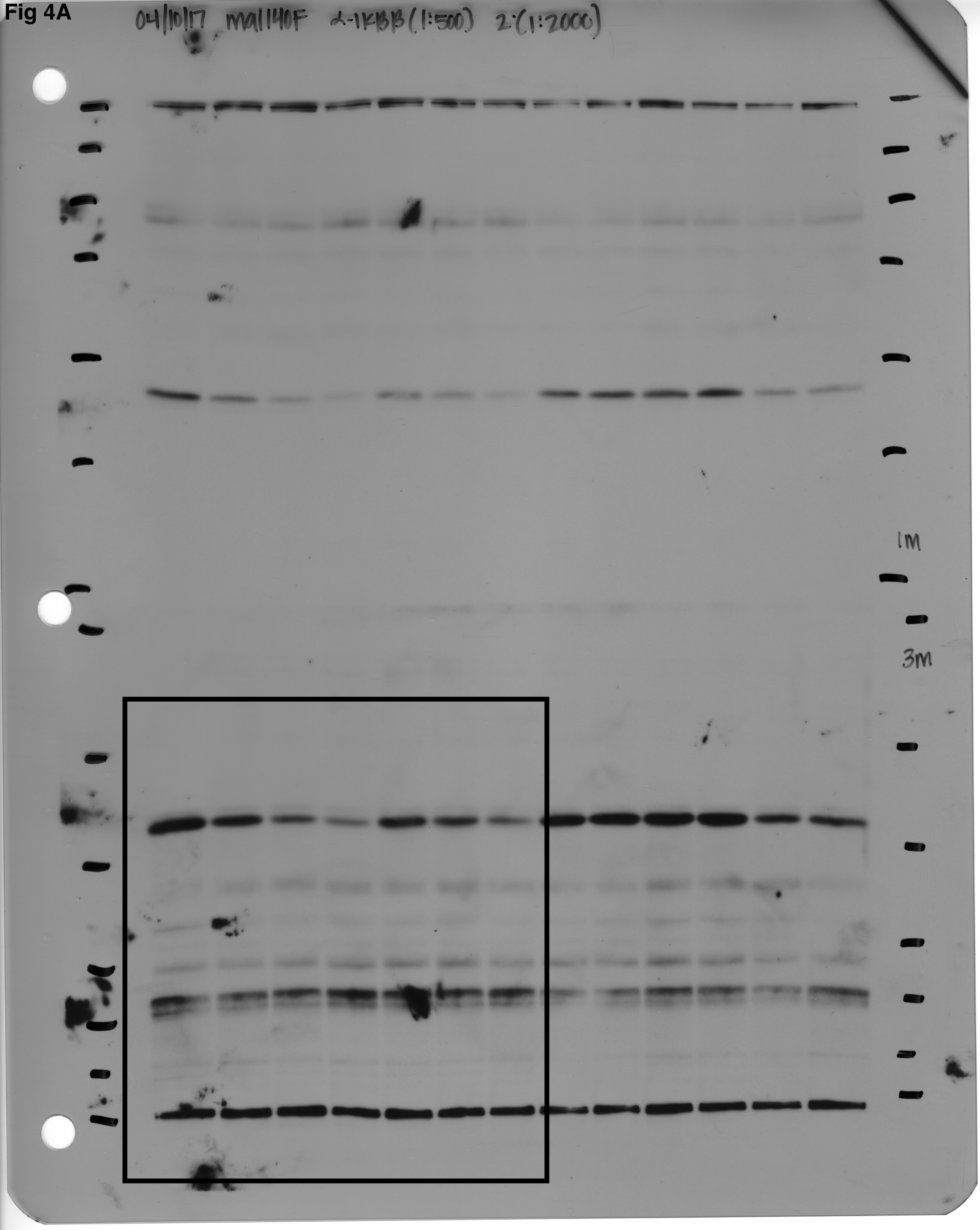

Fig 4A

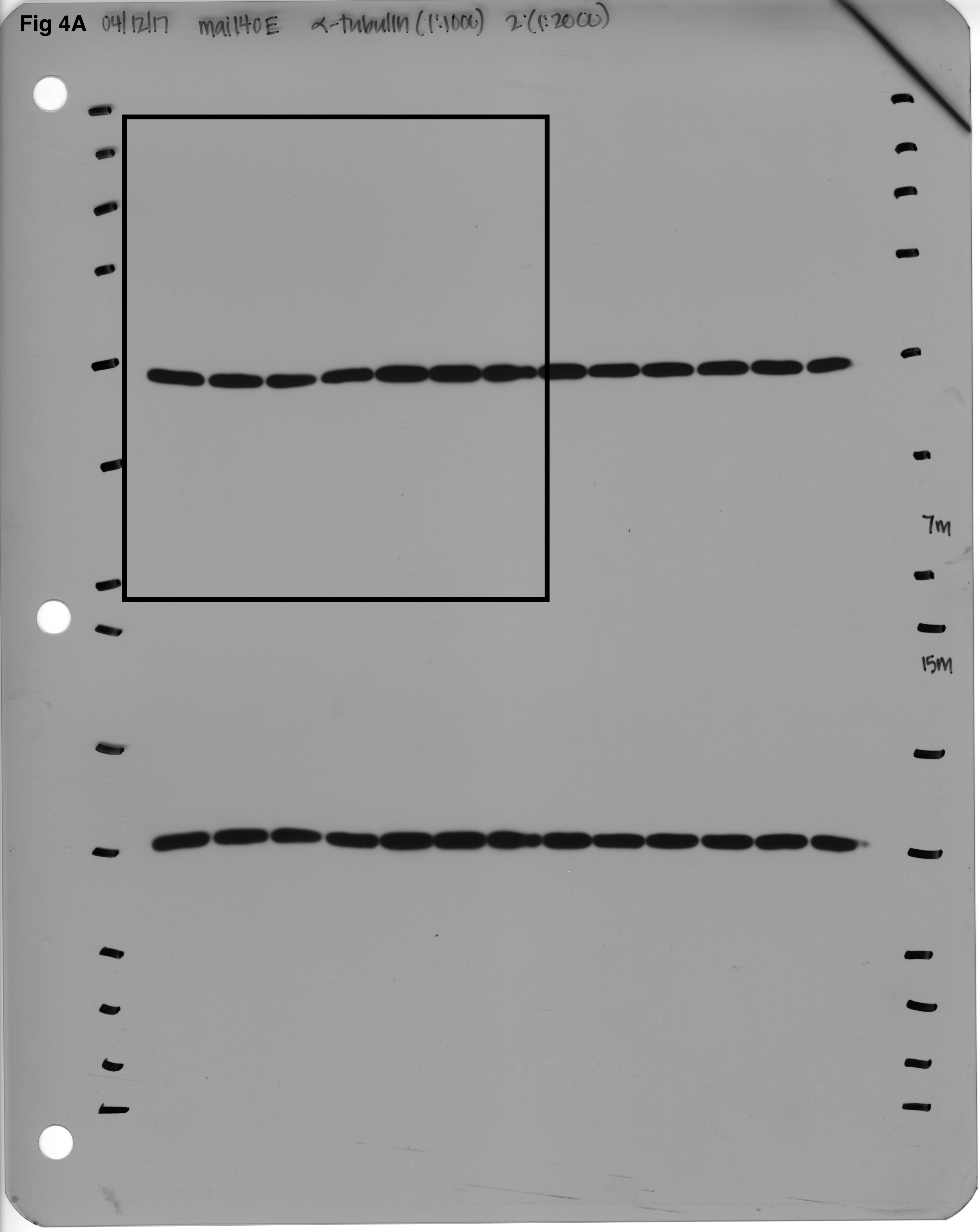

Fig 4C

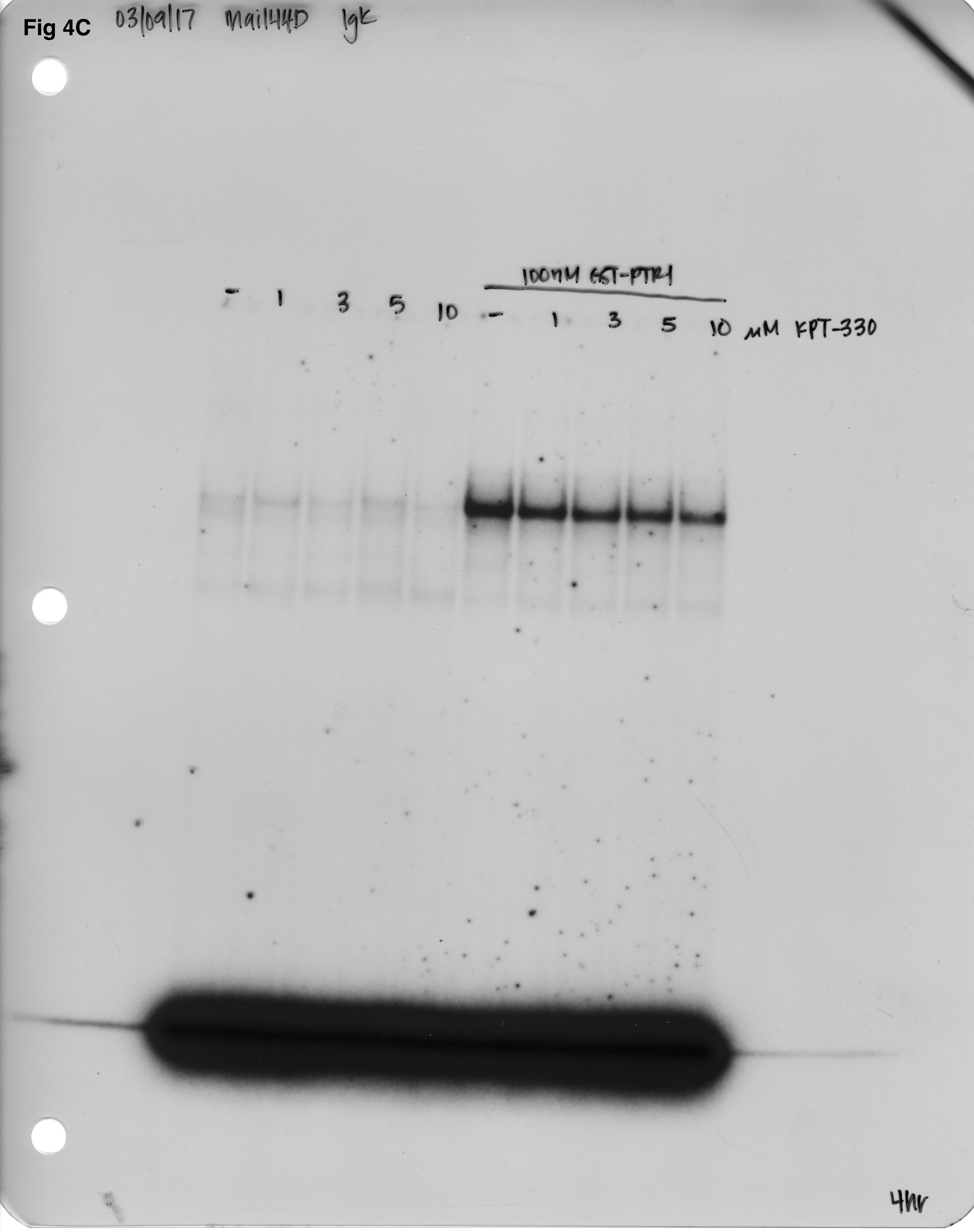

Fig 4C

03/09/17 mai144D oct-1

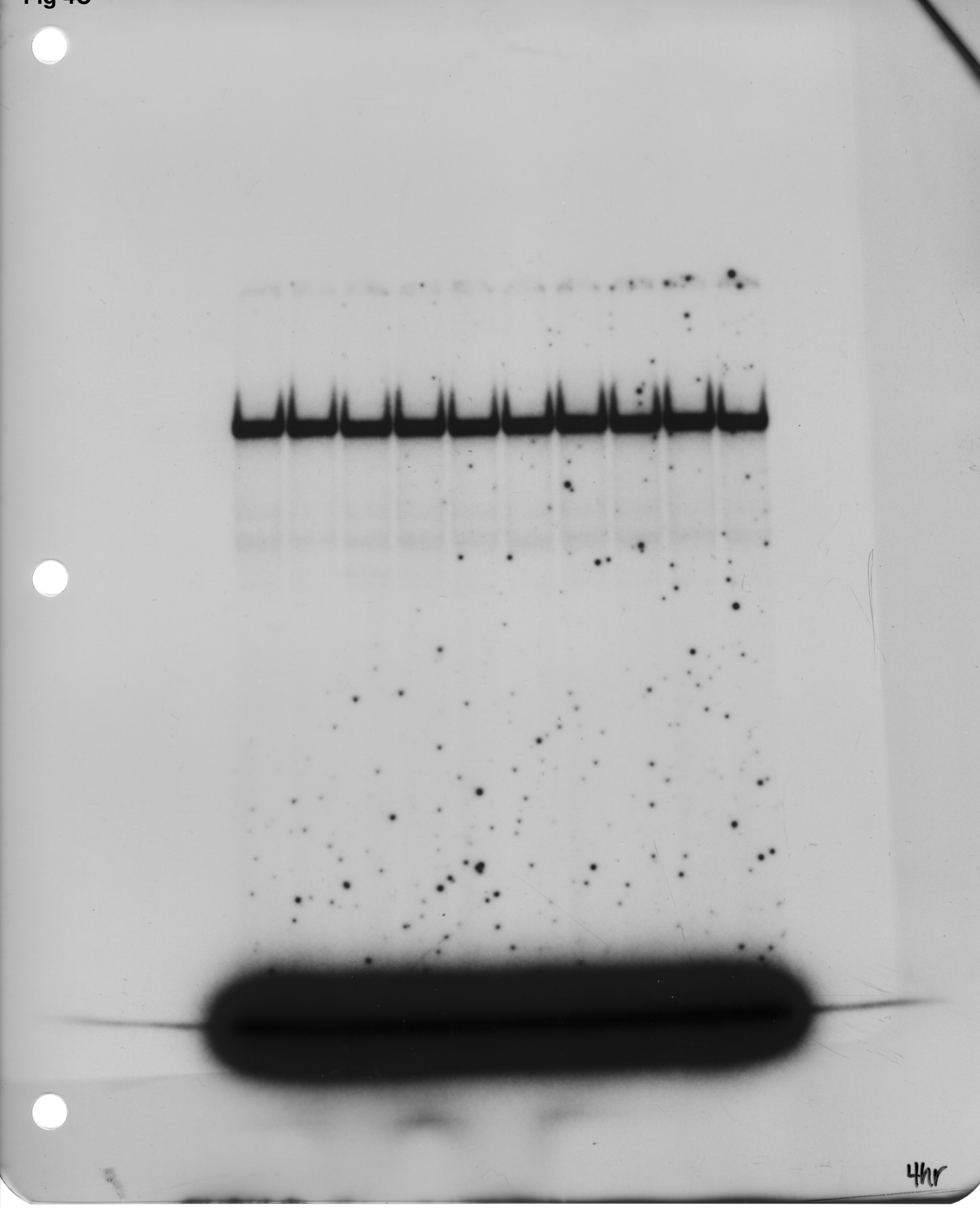

4hr

Fig 4D

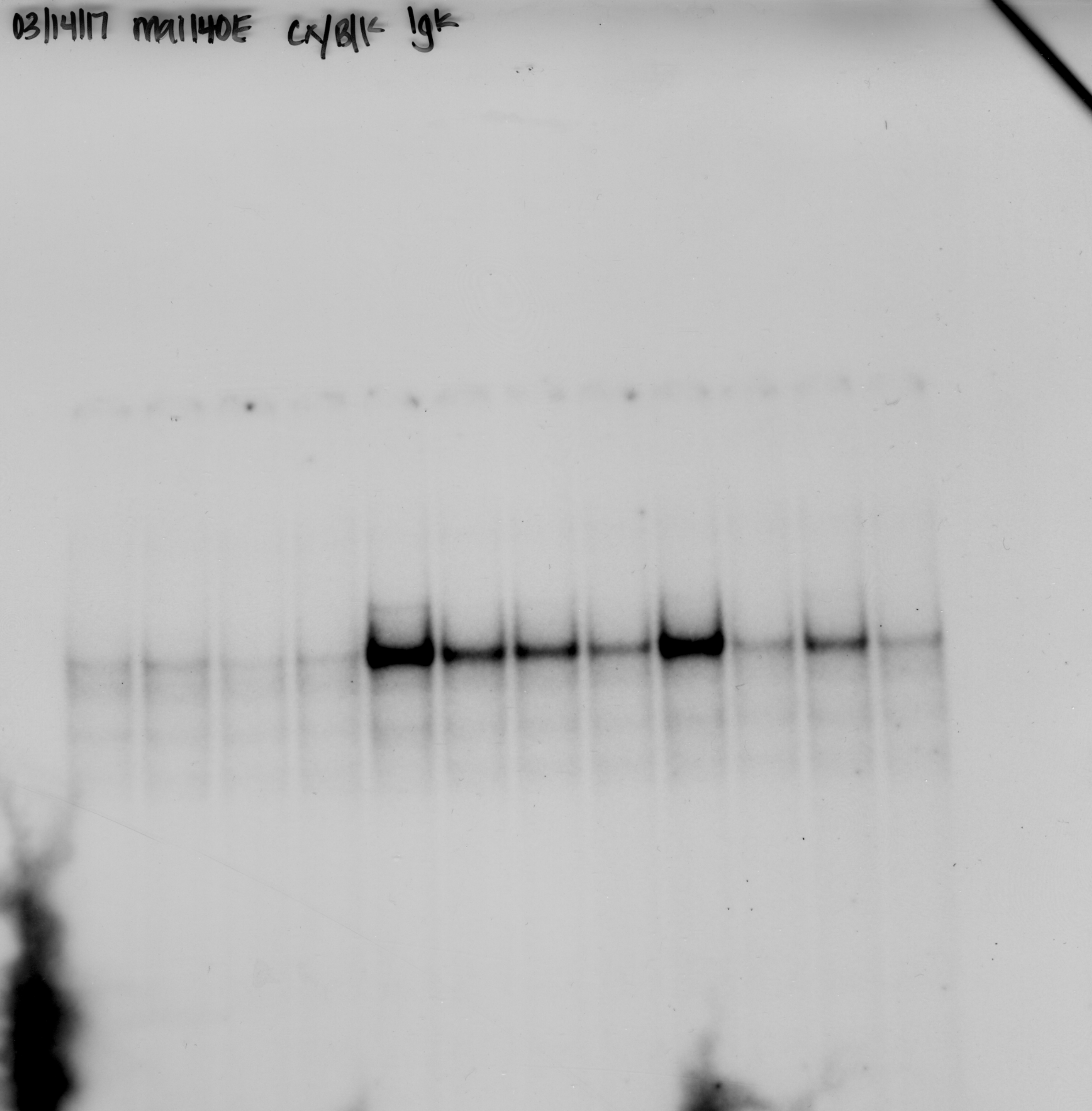

Fig 4D 03/13/17 Mai144E /Bort/KPT Oct-1

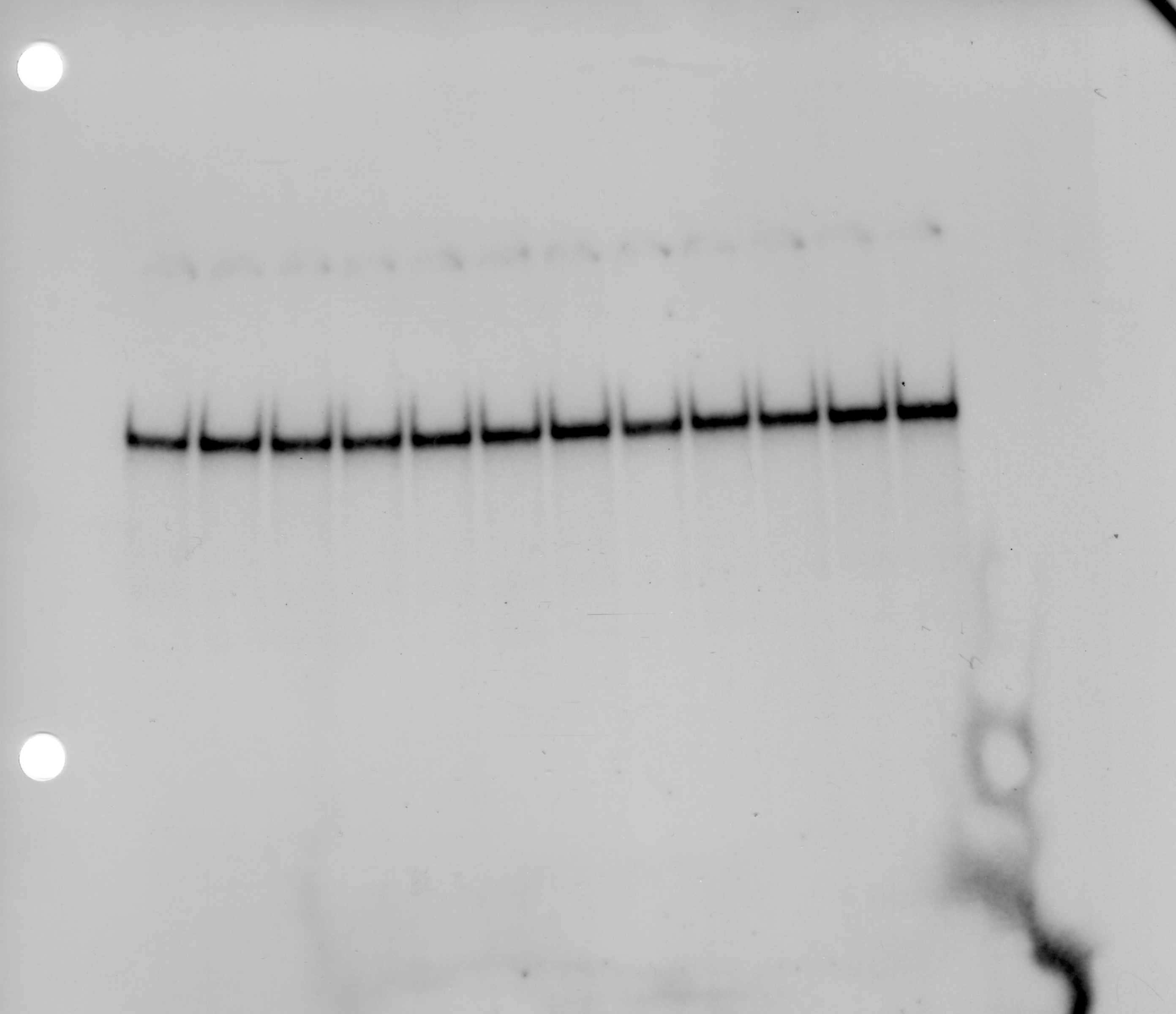

S1A Fig

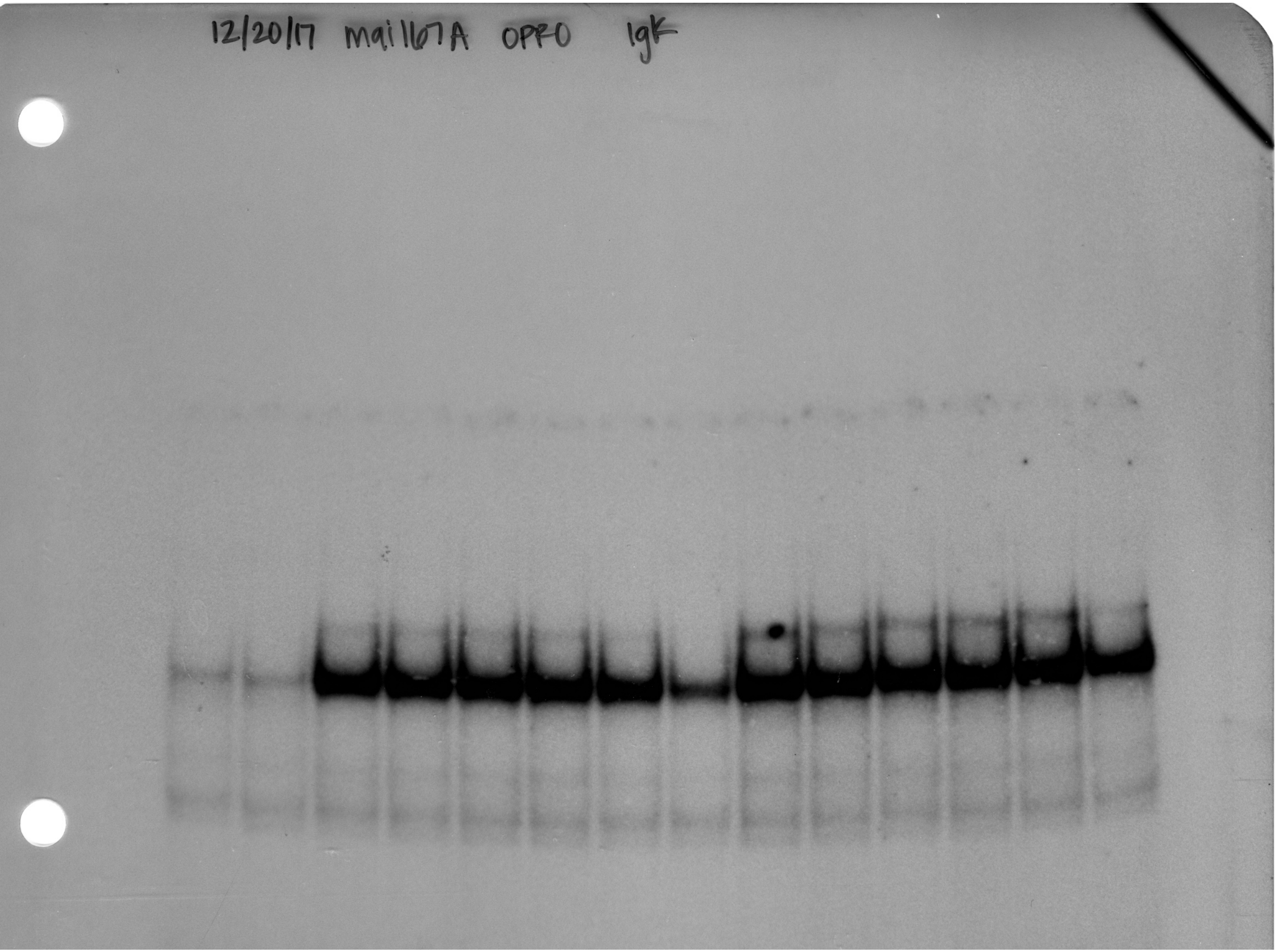

S1A Fig

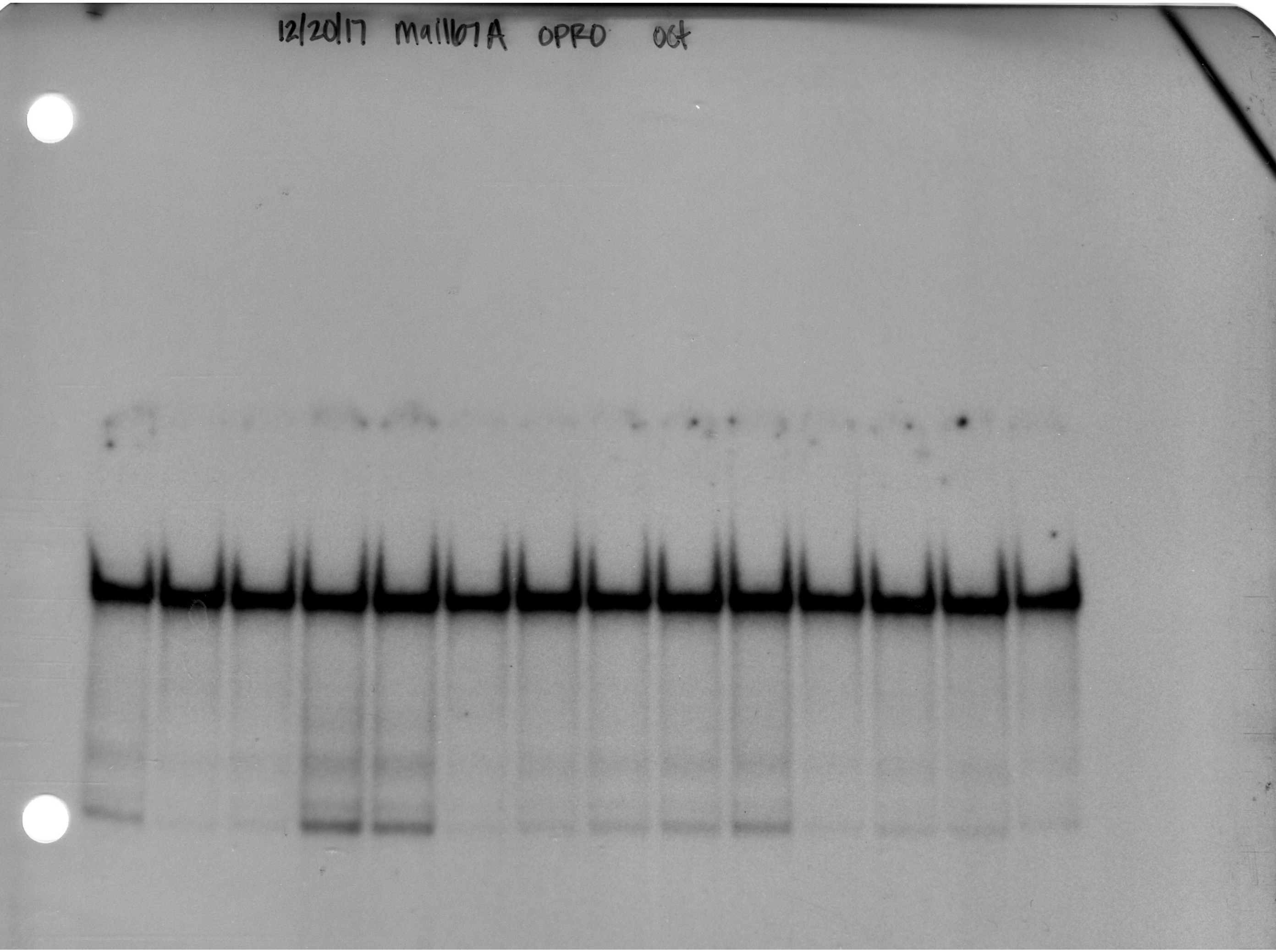

Supplement: S1 Raw images — (PDF) [file pone.0274704.s003.pdf]
